# Intracranial hemorrhages in patients with COVID-19: a systematic review of the literature, regarding six cases in an Amazonian population

**DOI:** 10.1055/s-0043-1772834

**Published:** 2023-11-30

**Authors:** William de Sousa Lima, Marcelo Henrique Pereira Soares, Eric Homero Albuquerque Paschoal, Joelma Karin Sagica Fernandes Paschoal, Fernando Mendes Paschoal, Edson Bor-Seng-Shu

**Affiliations:** 1 Universidade Federal do Pará, Faculdade de Medicina, Departamento de Neurologia do Hospital Universitário João de Barros Barreto, Belém PA, Brazil.; 2 Universidade de São Paulo, Faculdade de Medicina, Departamento de Neurologia do Hospital das Clínicas, São Paulo SP, Brazil.

**Keywords:** COVID-19, Intracranial Hemorrhages, Cerebral Hemorrhage, Hemorrhagic Stroke, COVID-19, Hemorragias Intracranianas, Hemorragia Cerebral, Acidente Vascular Cerebral Hemorrágico

## Abstract

**Background** Coronavirus disease 2019 (COVID-19) has emerged as a public health emergency worldwide, predominantly affecting the respiratory tract. However, evidence supports the involvement of extrapulmonary sites, including reports of intracranial hemorrhages.

**Objective** To describe six original cases and review the literature on intracranial hemorrhages in patients diagnosed with COVID-19 by molecular methods.

**Methods** A systematic literature review was performed on MEDLINE, PubMed, and NCBI electronic databases to identify eligible studies. Of the total 1,624 articles retrieved, only 53 articles met the inclusion criteria.

**Results** The overall incidence of intracranial hemorrhage in patients hospitalized for COVID-19 was 0.26%. In this patient group, the mean age was 60 years, and the majority were male (68%) with initial respiratory symptoms (73%) and some comorbidity. Before the diagnosis of hemorrhage, 43% of patients were using anticoagulants, 47.3% at therapeutic doses. The intraparenchymal (50%) was the most affected compartment, followed by the subarachnoid (34%), intraventricular (11%), and subdural (7%). There was a predominance of lobar over non-lobar topographies. Multifocal or multicompartmental hemorrhages were described in 25% of cases. Overall mortality in the cohort studies was 44%, while around 55% of patients were discharged from hospital.

**Conclusion** Despite the unusual association, the combination of these two diseases is associated with high rates of mortality and morbidity, as well as more severe clinicoradiological presentations. Further studies are needed to provide robust evidence on the exact pathophysiology behind the occurrence of intracranial hemorrhages after COVID-19 infection.

## Resumo

**Antecedentes** A COVID-19 emergiu como uma emergência de saúde pública em todo o mundo, proporcionando lesão principalmente do trato respiratório. No entanto, várias evidências apontam para acometimento de sítios extrapulmonares, incluindo relatos de hemorragias intracranianas.

**Objetivo** Descrever seis casos originais e revisar a literatura sobre hemorragias intracranianas em pacientes com diagnostico de COVID-19 por métodos moleculares.

**Métodos** A revisão sistemática da literatura foi feita nas bases de dados eletrônicas da MEDLINE, PubMed e NCBI para identificar os estudos elegíveis. Do total de 1.624 artigos recuperados, apenas 53 artigos preencheram os critérios de inclusão.

**Resultados** A incidência geral de hemorragia intracraniana nos pacientes internados por COVID-19 foi de 0,26%. A média de idade foi de 60 anos, e a maioria dos pacientes era do sexo masculino (68%) com sintomas respiratórios iniciais (73%) e alguma comorbidade. Antes do diagnóstico de hemorragia, 43% estavam em uso de anticoagulantes, 47,3% destes em doses terapêuticas. O compartimento mais acometido foi o intraparenquimatoso (50%), seguido do subaracnoideo (34%), intraventricular (11%) e subdural (7%). Houve predomínio de topografias lobares sobre as não-lobares. Hemorragias multifocais ou multicompartimentais foram descritas em 25% dos casos. A mortalidade geral nos estudos de coorte foi de 44%, enquanto houve alta hospitalar em cerca de 55% dos pacientes.

**Conclusão** Apesar da associação incomum, a combinação dessas doenças está relacionada com altas taxas de mortalidade e morbidade, bem como apresentações clínico-radiológicas mais graves. Mais estudos são necessários para oferecer evidências robustas sobre a fisiopatologia exata por trás da ocorrência de hemorragias intracranianas após infecção por COVID-19.

## INTRODUCTION

A few months after the outbreak of the novel coronavirus disease 2019 (COVID-19), in March 2020, the World Health Organization (WHO) declared a pandemic. Within just 2 years, cumulative COVID-19 cases had reached 455 million, with a death toll of around 6 million worldwide. 1 The β-coronavirus, whose full name is severe acute respiratory syndrome coronavirus 2 (SARS-CoV-2), causes an infection predominantly of the lower and upper respiratory tract, but there is evidence of involvement of extrapulmonary sites: cardiovascular, central nervous system, gastrointestinal, renal, hepatic, hematologic, and cutaneous. 2 3

Neurological manifestations of COVID-19 include headache, dizziness, altered level of consciousness, hyposmia, hypogeusia, cerebrovascular diseases (CVDs), polyneuropathies, ataxia, and epileptic seizures. Although CVD events are among the least common manifestations, they are one of the most serious and fatal. 4 5

Imaging findings in the patients with neurological symptoms include numerous disorders, with stroke being the most prevalent and dangerous. Hemorrhagic stroke has even higher mortality and symptom severity than ischemic stroke. 6 7 8 Intracranial hemorrhages (ICHs) can be classified into five broad categories: intraparenchymal hemorrhage (IPH); intraventricular hemorrhage (IVH); epidural hematoma (EDH); subdural hematoma (SDH); and subarachnoid hemorrhage (SAH). 9 10

The mechanisms behind neurological involvement, although not yet fully clear, include direct and indirect damage caused by the virus upon invasion of the central nervous system (CNS), involving both hematogenous and retrograde neuronal pathways in the invasion of olfactory neurons. 11 The angiotensin II-converting enzyme (ACE2) receptor plays a key role in the mechanism of cell invasion and breakdown of the blood-brain barrier (BBB). Respiratory epithelial cells, neurons and glial cells express ACE2 receptors in abundance. 12 Nevertheless, there are also mechanisms of indirect injury mediated by the systemic inflammatory syndrome promoted by the storm of proinflammatory cytokines and chemokines, also implicated in the breakdown of the BBB. 13

In general, endothelial dysfunction leads to a systemic prothrombotic state related to high levels of proinflammatory cytokines and also angiotensin II. 14 Findings of abnormalities on coagulation tests and high serum levels of D-dimer, ferritin, and LDH corroborate this hypothesis. 15 16 Thus, there is a greater tendency for ischemic than hemorrhagic events, and the somewhat paradoxical occurrence of these intracranial hemorrhages might be attributed to blood pressure dysregulation and BBB breakdown. 14

Thus, the aim of this study was to report six original cases of COVID19-related cerebral hemorrhages in patients who presented at a health care facility. Furthermore, the current study, as part of a systematic review, aimed to assess the existing evidence within the literature concerning cases of COVID-19 (confirmed through the real-time polymerase chain reaction [RT-PCR] method) and their potential correlation with intracranial hemorrhage. Additionally, the study aimed to delineate the demographic, clinical, and radiologic characteristics associated with these cases.

## METHODS

### Case series

Six patients with cerebral hemorrhages related to COVID-19 infection were selected. These cases were observed between 1st May 2020 and December 28th, 2020 at the Air Force Hospital of Belém. The patients had a confirmed diagnosis of COVID-19 through RT-PCR testing, and a diagnosis of ICH was established based on clinical-radiological aspects. Neuroimaging was done in all patients. After reviewing the neuroimaging reports for these patients, they were found to have documented radiographic evidence of hemorrhage. Neuroimaging for these patients was reviewed by a fellowship-trained neuroradiologist to verify the presence and type of hemorrhage. This diagnosis was further corroborated by neuroimaging tests, which included computed tomography (CT) or magnetic resonance imaging (MRI).

The six patients in this case series were not encompassed within the scope of the systematic review conducted in this study.

### Literature search strategy

A comprehensive, systematic search of the literature published between December 19, 2021, and May 7, 2022, held on the MEDLINE, PubMed, and NCBI electronic databases was conducted using the following search terms: ( *hemorrhagic encephalopathy* ) OR ( *intracranial bleeding* ) OR ( *subarachnoid hemorrhage* ) OR ( *subdural hemorrhage* ) OR ( *intracranial hemorrhage* ) OR ( *hemorrhagic stroke* ) OR ( *cerebral hemorrhagic complication* ) OR ( *cerebral hemorrhage* ) AND ( *SARS-CoV-2 virus* ) OR ( *SARS CoV 2 virus* ) OR ( *2019-nCoV* ) OR ( *COVID-19* ) OR ( *2019 novel coronavirus* ).

### Eligibility criteria

The search was limited to articles written in English. Articles identified by the initial search strategy were independently evaluated by two authors (WL and MP) according to the inclusion criteria: involving patients with COVID diagnosed by RT-PCR, a confirmed diagnosis of ICH, description of the cases with individual demographic characteristics, clinical-radiological aspects, interventions, and outcomes. Articles which were duplicates, those that had only the abstract available or were editorial letter articles, as well as those whose full-text was not in English, and those that involved pediatric patients (age < 18) and patients with predominantly non-spontaneous hemorrhages were excluded.

### Study selection and quality control

The Covidence systematic review software (Veritas Health Innovation, Melbourne, Australia) was used to import all titles and abstracts of the articles identified and remove duplicate records. Potentially eligible articles were identified by screening the titles and abstracts. The full texts of the studies selected were then thoroughly reviewed for quality control by two authors (WL and MP) using the Newcastle-Ottawa scale, and the eligibility of each study was determined. Any disagreements between the investigators were resolved by consulting with the corresponding author (FP) ( Supplementary Material 1 https://www.arquivosdeneuropsiquiatria.org/wp-content/uploads/2023/10/ANP-2022.0230-Supplementary-Material-1.docx ).

### Data extraction

The following information was collected from each study reviewed: surname of the first author and year of publication, study design, sample size, demographic characteristics, comorbidities, number of patients with hemorrhagic events in COVID-19 hospital admissions, time interval from admission/initial symptoms to radiological diagnosis, initial laboratory findings, antithrombotic therapy prior to onset of hemorrhagic event, type of ICH, clinicoradiological scales applied on admission and/or discharge, mortality rates, and discharge outcomes. Neuroimaging findings were divided into three major types: intraparenchymal hemorrhage (IPH), subdural hematoma (SDH), and subarachnoid hemorrhage (SAH). Additionally, regarding the hemorrhage distribution, we also classified three subtypes: focal intracerebral hemorrhage (FICH), multifocal intracerebral hemorrhage (MFIH), and multicompartmental hemorrhage (MCH). 9 10 17

### Synthesis of results

The synthesis of the data was performed with the aid of the Covidence and Excel (Microsoft Corp., Redmond, WA, USA) programs, where data extracted were compiled into tables with their respective categories. Primarily, the relevant findings on eligible cohort and case series studies reporting ICH in COVID-19 hospitalizations were presented in the form of a summary table ( Supplementary Material 2 , **Table 1** ) accompanied by a narrative description. A concise overview of the attributes of the six patients featured in this case series has been incorporated into Supplementary Material 2 , **Table 1** . The remaining case reports identified by the search were subsequently compared against our original case reports and stratified into additional tables by similar hemorrhagic events ( Supplementary Material 2 , **Tables 2–4** ).

## RESULTS

### Case series

The cerebral hemorrhage causes identified in the six selected patients were as follows: IPV/IVH in two cases, both accompanied by indications of intracranial hypertension and uncus herniation; one case with SDH featuring mass effect on the right frontal, temporal, and parietal lobes, alongside indications of intracranial hypertension; two cases with CVT/IPH; and one case with IS/IPH. Detailed clinical and demographic attributes of the patients within this case series can be found in Supplementary Material 2 .

### Study identification and eligibility

**Figure 1:**
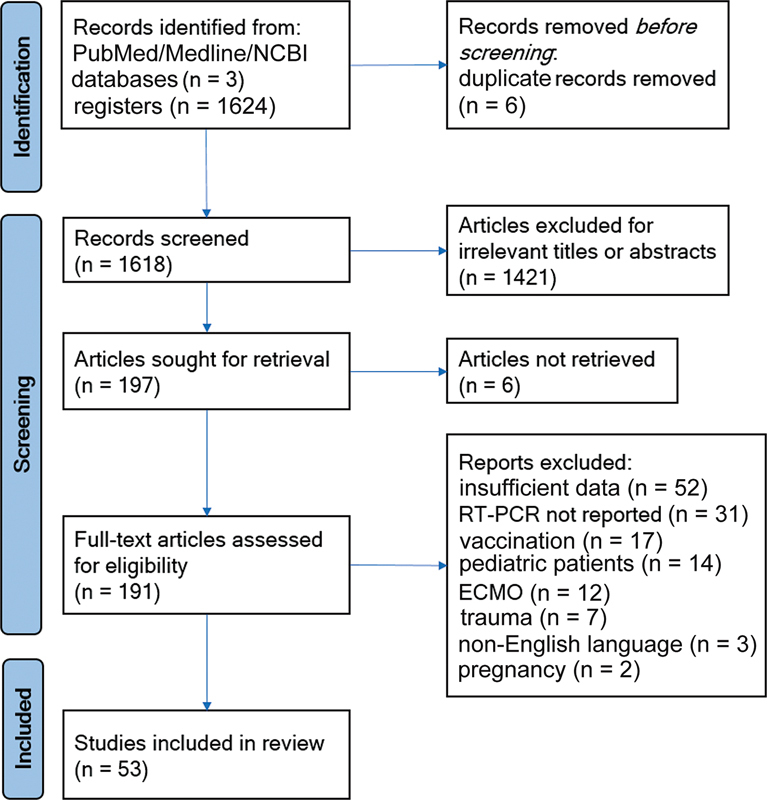
PRISMA flow diagram of included articles. Source: PRISMA 2020 statement: an updated guideline for reporting systematic reviews. Page MJ, McKenzie JE, Bossuyt PM, Boutron I, Hoffmann TC, Mulrow CD, et al. BMJ. 2021;372:n71. DOI: 10.1136/bmj.n71. Available from: http://www.prisma-statement.org/ .

Of a total of 1,624 articles retrieved in the literature search up to March 2022, 6 duplicate studies were removed, and 1,618 articles retained for screening of title and abstract. After exclusion of 1,421 non-relevant studies, 197 studies were retrieved, of which an additional 144 were subsequently excluded for the reasons presented in Figure 1 . The selection process resulted in a final total of 53 articles for inclusion in the review.

### Characteristics of studies reviewed

Considering 22 cohort selected articles, the prevalence of hemorrhagic cerebrovascular events among patients with COVID-19 was described in Supplementary Material 2 , along with epidemiological data. A total of 31 case reports and case series articles were included to report the sex, age, comorbidities, initial symptoms, diagnostic methods, radiological findings, treatment, and outcome. ( Supplementary Material 2 )

### Data synthesis

#### Incidence of ICH in COVID-19 patients with positive RT-PCR

For the cohort studies, the overall incidence of ICH was ∼ 0.26% among 168,703 patients from the 22 studies evaluated. Regarding range, the studies with the lowest and highest incidence reported 0.06% in 1,661 cases 5 and 23.7% in 80 patients 18 assessed, respectively.

### Demographic aspects of patients with COVID-19 and ICH

#### Age and sex

For the overall 41 articles involving a total 414 cases, patients had a mean age of 60 years and were predominantly male (67%). Among the cohort studies only, patients were 73% male and had a mean age of 62 years, while the cohorts of only 4 articles 19 20 21 22 had a mean age of < 60 years ( Supplementary Material 2 , **Table 1** ). However, for the reports and case series, patients had a mean age of 54 years and 60% were male.

#### Comorbidities

Only half of the cohort studies reported information on comorbidities 17 18 19 20 22 23 24 25 26 27 28 ( Supplementary Material 2 , **Table 1** ). Hypertension and type 2 diabetes mellitus (DM2) were cited in all such articles, with prevalence ranges of hypertension of 37 to 100%, DM2 11 to 49.4%, and dyslipidemia 8.3 to 67%. The rates of atrial fibrillation 23 25 27 28 were 5.2 to 31.8%, tobacco use 18 20 24 25 26 5.3 to 66.6%, coronary disease 19 28 12.1 to 38%, and congestive heart failure 23 25 17.1 to 24.7%.

Previous bleeding events 18 19 23 25 ranged from 12.5% to 18%. Cancer was cited in only 2 articles, 17 22 affecting 14 to 33% of the samples investigated. Other comorbidities, such as chronic kidney disease, 23 obesity, 19 alcoholism, 25 and previous myocardial infarction, 17 were present in only one cohort each.

#### Anticoagulation prior to ICH onset

Twenty-five articles, including cohort studies, case series, and case reports, reported administration of some form of anticoagulation in 43% of the 385 patients before the diagnosis of cerebral hemorrhage. Of these, 167 patients (47.3%) used therapeutic doses of anticoagulants, and antiplatelet agents were used in 4% of the 385 cases.

#### Initial clinical presentations

Out of the 125 COVID-19 cases, 73% initially had respiratory symptoms before the cerebrovascular event, while the remainder had early neurological symptoms. Reported symptoms included sudden severe headache, aphasia, hemiparesis, seizures, altered level of consciousness, and coma.

The time elapsed between the initial symptoms and diagnosis of the event was reported for 131 cases, revealing an average period of 10 days. However, some studies, 26 28 29 30 involving a total of 61 cases, described the time between patient admission and diagnosis of the event, revealing a mean interval of 13 days. The National Institutes of Health Stroke Scale (NIHSS) was used in 74 of the cases reviewed, with a mean score of 19.7 while the Glasgow Coma Scale (GCS) was used in 90 patients, with scores averaging 7.7.

#### In-hospital events during hospitalization

Six articles reported other events during the hospital stay in 211 patients. 18 19 25 29 31 32 The complications presented were acute kidney injury (44%), sepsis (30%), myocardial injury (14%), urinary tract infection (12%), deep vein thrombosis (7%), hepatic failure (5%), and venous thromboembolism (3%). 18 19 25 29 31 32

#### Characteristics of ICH

Of the 561 cases included in the review, descriptions of ICH neuroradiological features were provided for 342 (61%). The most frequent presentations were IPH (50%) and SAH (34%), followed by FICH (17%), MFH (15%), microhemorrhages (12%), IVH (11%), MCH (10%), hemorrhagic conversion (9%), and SDH (7%). Analyzing the cohort studies only, the distribution of hemorrhage types differed: IPH (45%), SAH (27%), microhemorrhages (16%), MFH (15%), hemorrhagic conversion (10%), FICH (9%), SDH (9%), MCH (8%), and IVH (7%) ( Supplementary Material 2 ).

#### Intraparenchymal hemorrhage

**Figure 2:**
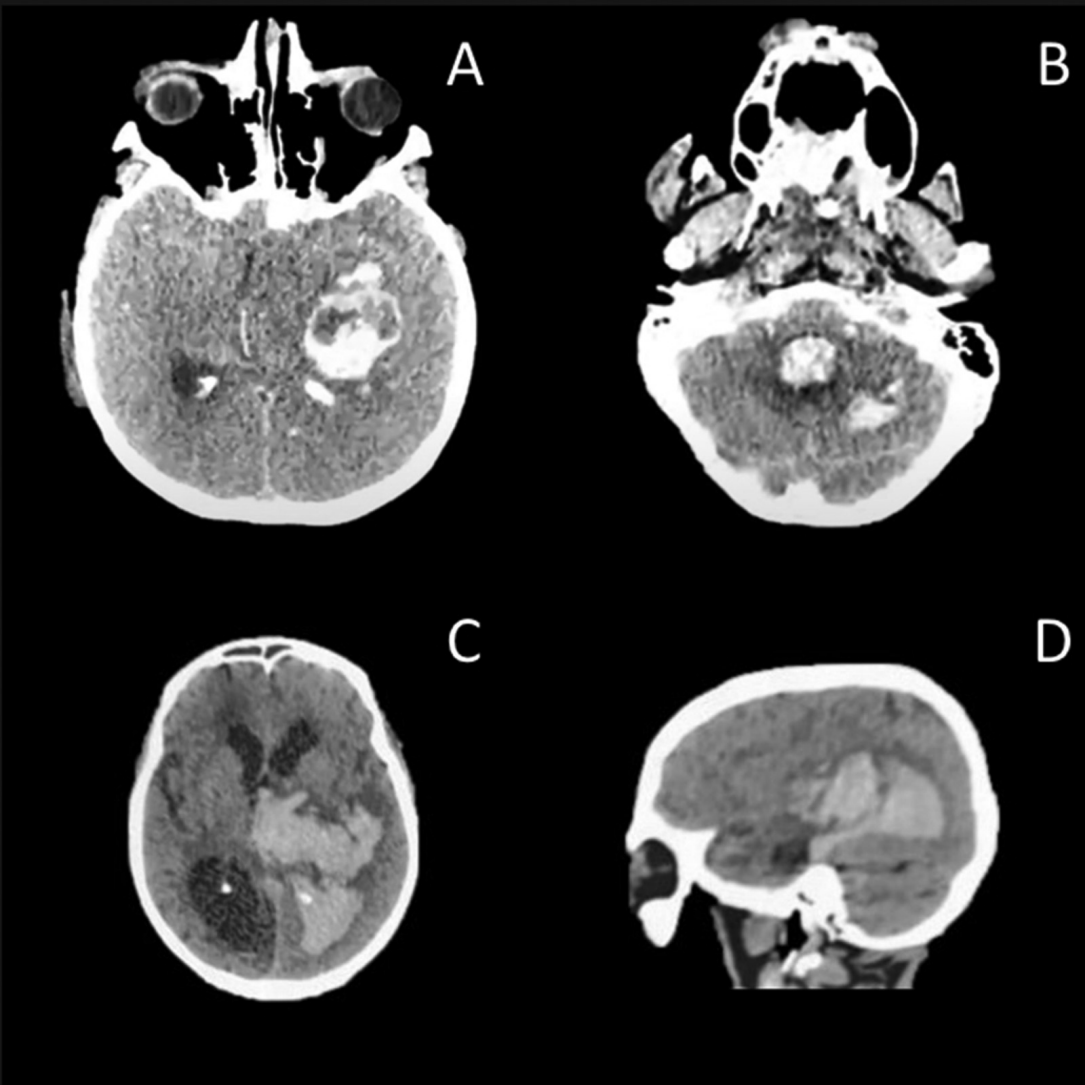
Intraparenchymal hemorrhages. (A-B) Patient 1: 66 years-old male presenting with decreased level of consciousness. Axial non-contrast CT images ( **A** ) showing an intraparenchymal hemorrhage in temporal lobe region with left to right midline shift and ( **B** ) extension to fourth ventricle in association with left cerebellar hemorrhage. Patient 2: 59-year-old female presenting with seizures and decreased level of consciousness. Axial and sagittal non-contrast CT images ( **C-D** ) demonstrating an extensive intraparenchymal hemorrhage involving left basal ganglia and temporoparietal areas with a left to right midline shift and extension to posterior horn of left lateral ventricle, associated with ventricular dilatation of posterior horns of lateral ventricles.

IPH was reported in 36 articles, representing 170 patients. The location of the IPH was supratentorial in 66% of cases and infratentorial in 14%, while the remainder had a non-specified location. Additionally, lobar locations accounted for 32% and non-lobar for 24% of cases. Supratentorial hemorrhages were described in 99 cases with the following site distribution: lobar (51%), cortical (28%), basal ganglia (12%), and thalamic (5%) ( Supplementary Material 2 ) ( Figure 2 ).

Some cohorts specified IPH case characteristics in which, in this patient group, up to 75% used anticoagulants, the majority were male (81%), initial symptoms were respiratory (81%), and mean age was 61 years. 18 19 22 24 26 27 The volume of the IPH was measured in 54 patients, with a mean value of 37.1 cm ^3^ (0.4–125 cm ^3^ ). Moreover, the mean ICH score for 106 patients was 2.46. (0–5). 23 26 27 29

#### Multicompartmental hemorrhages

A total of 16 studies including a total of 35 patients with MCH were identified. The combinations of hemorrhage locations included IPH/SAH/IVH ( *n* = 11), IPH/SAH ( *n* = 10), IPH/SAH/SDH ( *n* = 10), SDH/SAH ( *n* = 2), and IPH/SDH ( *n* = 1). Some cohorts specified MCH case characteristics, revealing that patients had the same profile of IPH group, and more than half (53%) of these patients were on anticoagulation agents ( Supplementary Material 2 ).

#### Subarachnoid and subdural hemorrhages

Of a total of 118 cases reported in 34 articles, secondary or undetermined SAH was the most prevalent cause, representing ∼ 75% of cases, while aneurysm and arterial dissection represented 18% and 7% of patients, respectively. Six cohort studies described SAH features, according to which up to 86% of patients were on anticoagulation agents, most had initial respiratory symptoms (62%), and the mean age was 62 years. 17 18 19 21 26 27 When including case series and reports, the mean age was 54 years, 81% had respiratory onset, and 68% used anticoagulants.

Only 25 patients in 8 studies reported SDH; these cases were either isolated ( *n* = 20) or associated with multicompartmental hemorrhage ( *n* = 5). Up to 70% of these patients were male and the mean age was 74 years, while 30% used anticoagulants ( *n* = 20). ( Supplementary Material 2 )

The aneurysmal arteries reported were the posterior inferior cerebellar artery (10%), posterior cerebral artery (10%), anterior choroidal artery (10%), anterior communicating artery (10%), middle cerebral artery (5%), and ophthalmic artery (5%). Moreover, the dissecting arteries were vertebral artery (33%), posterior inferior cerebellar artery (22%), anterior communicating artery (11%), middle cerebral artery (11%), posterior cerebral artery (11%), and internal carotid artery (11%).

#### Hemorrhagic conversion

**Figure 3:**
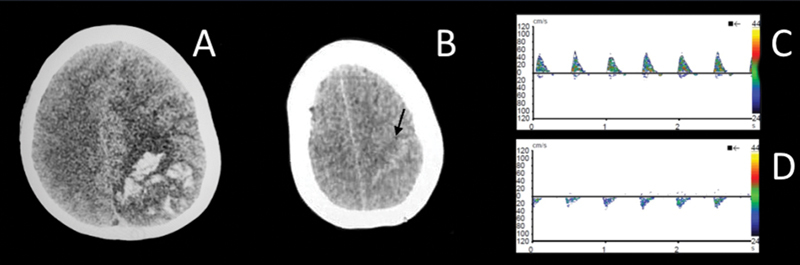
Hemorrhagic transformation of cerebral venous thrombosis (CVT). ( **A-D** ) Patient 4: 39-year-old female presenting with severe progressive headache. Axial non-contrast CT images ( **A** ) demonstrating the “cord sign” (black arrow) indicating thrombosis of cerebral cortical veins; and ( **B** ): intraparenchymal hemorrhage in left parietal lobe with left-to-right midline shift. Transcranial doppler ( **C-D** ) shows spectral image with spikes on middle cerebral artery monitoring, indicating cerebral circulatory collapse.

Thirty-two patients were identified, in 10 separate articles, who suffered a hemorrhagic conversion of some kind during a COVID-19 infection. Ischemic stroke (IS) was the main cause of these hemorrhages ( *n* = 29) ( Figure 3 ), followed by cerebral venous thrombosis (CVT) ( *n* = 3).

Furthermore, among the case reports, 6 out of the 8 patients had respiratory symptoms, with time from onset to diagnosis of 0 to 21 days, while the remaining 2 cases had a typical acute stroke presentation. Only one patient was managed surgically, and three patients had a poor prognosis with death or multiple organ failure, but none had CVT ( Supplementary Material 2 , **Tables 1** and **4** ).

### Interventions for ICH

Interventions targeting ICH were reported in 10 cohort studies involving a total of 269 cases 17 18 19 20 22 24 25 26 27 33 ( Supplementary Material 1 , **Table 1** ). Surgical management was performed in 29 patients (10.7%), 16 of whom had external ventricular shunt (EVD), 5 hematoma drainage with decompressive craniectomy, 2 aneurysm embolization by coiling or flow deviation, 2 had invasive monitoring of intracranial pressure (ICP), and the remaining reports were unclear on the type of surgical approach employed. Management solely by intensive care measures occurred in 45 patients (16.7%). However, the use of specific measures to control ICP or hemorrhage was not specified.

### Mortality in COVID-19 patients with ICH

Among the cohort studies, 11 articles described the mortality rate, which ranged from 0 to 84.6% 17 18 19 20 22 23 24 25 26 27 28 ( Supplementary Material 2 ). The overall mortality rate in these studies was 44%, for a total of 313 patients out of 114,706 cases hospitalized with COVID-19. The mortality rate from cerebrovascular hemorrhagic events in hospitalized patients with a confirmed diagnosis of COVID-19 by the RT-PCR method was 0.12%. When case series studies and case reports were included, a total of 427 patients were obtained with an overall mortality rate of 46.3%.

### Outcomes reported

Other outcomes were reported in 7 cohort studies, 17 18 20 22 24 25 27 with descriptions of hospital discharge (non-routine, to home or to rehabilitation) in 57.5% of a total of 245 patients. In addition, modified Rankin Scale (mRS) scores at discharge were variably reported and incomplete for the majority of the studies reviewed. Only 4 articles 20 22 24 26 mentioned a poor prognosis at discharge (mRS > 3), in 69.5% of a total of 23 patients who were evaluated at discharge with this scale ( Supplementary Material 2 ). Including case series and case report studies, hospital discharge was reported in 53.5% out of a total of 327 patients, while there was a poor prognosis (mRS > 3) in 73% of a total of 110 patients ( Supplementary Material 2 ).

## DISCUSSION

Several epidemiological studies have reported a significant reduction in hospital admissions involving stroke cases of all types during the first wave period. The decrease in admissions reported by these studies ranged from 12 to 45.6%. 34 35 36 37

There was a more significant reduction for transient ischemic attack (TIA) and IS admissions, although no significant decrease for hemorrhagic stroke cases, possibly explained by the low incidence of this type of event. 35 37 38 39 Other authors found similar results, but reported a significant decrease in hemorrhagic stroke cases. 36 37 40

To investigate this impact, a large observational study involving 187 major stroke centers in 40 countries assessed the impact of the COVID-19 pandemic on hospital admissions for ischemic and hemorrhagic stroke, as well as for the volume of mechanical thrombectomy. A significant global decline was reported in all stroke care indicators during the early COVID-19 pandemic, including a drop in the volume of mechanical thrombectomy procedures (12.7%), overall stroke admissions (19.2%), IS/TIA admissions (15.1%), and of ICH hospitalization cases (11.5%). 41

Possible explanations for this phenomenon include the cancellation of elective surgeries due to the pandemic, leading to a decrease in perioperative stroke. The lockdown situation may have been a factor improving medication adherence, which can lead to a decrease in cerebrovascular diseases. 41

These findings are consistent with the reported increase of in-hospital mortality for stroke in some studies, 35 37 42 43 44 while other studies also found a more marked increase for ICH. 17 23 27 28 33 In addition, there is a contradiction if the pandemics caused a change in the proportion of moderate/severe stroke (NIHSS scale > 5), with some studies reporting an increase, 36 42 while others found no significant change. 34 37 39

However, in an observational study of patients aged > 80 years, it was noted that the onset of stroke did not increase the risk of death, and those who survived COVID-19 and an acute stroke had similar outcomes to those without this complication. Active smoking, previous history of stroke, along with a low BMI were identified as significant risk factors for cerebrovascular complications in this age group. 45 Among our original cases reported, the 2 patients aged > 70 years had a good prognosis, including complete functional recovery after the stroke event.

Some large meta-analyses involving more than 60,000 patients reported the incidence of CVDs among the group of SARS Cov2-positive admissions, where rates ranged from 1.2 to 1.4% for general CVDs 46 47 48 and from 0.2 to 0.3% for ICH. 46 47 Additionally, CVD in these patients was associated with more severe infectious disease and an ∼ 5-fold increased mortality, 46 48 while a severe infection increased the risk of CVD and ICH by ∼ 3-fold and 7-fold, respectively. The reported mortality rates for ICH and IS were 44.7% and 36.2 to 38%, respectively. 47 49

Although these meta-analyses did not include solely RT-PCR confirmed cases, slightly different results were found in two other meta-analyses which selected only patients confirmed by this method. Slightly higher incidence rates were found for CVD (1.5%) and ICH (0.15–0.7%), 50 51 with higher mortality rates for ICH (48.6%). 50 However, a lower mortality rate was reported for IS (22.8%). 51 In the present review, based on data from 22 cohorts and a total of 168,703 cases, the ICH incidence was 0.26%, a rate consistent with the studies cited. The mortality rate was 44%, calculated using data from 11 cohorts including a total of 114,706 cases. When compared these with our six cases of positive RT-PCR, an even higher mortality rate of 50% was found.

### Clinical-radiological aspects

In general, the most common neurological symptoms described in COVID-19 patients are headache, altered level of consciousness (ALC), dizziness, ageusia and anosmia, while other less common symptoms reported include visual impairment, CVD, seizures, occipital neuralgia, ataxia, tremor, and tics. 4 5 27 Severe infections were more likely in the presence of CVD and ALC 27 and to be reported in hypertensive patients, who were older, had fewer typical symptoms, and were more likely to develop neurological manifestations, especially acute CVD. 4

Several of the articles reported the time between admission and neuroimaging, with mean values ranging from 11 to 29 days. 22 26 28 52 The interval between the onset of symptoms and diagnosis was similar, lying within the range 2 to 29 days. 19 24 27 29 52 53 54 55 Acute stroke signs were the initial manifestation of COVID-19 in only 11 to 44% of admissions. 26 29 32 44 50 52 54 56 Our 6 cases are in agreement with the literature as presenting a 10 days median time and 33% of neurologic symptoms onset. At admission, the main neurologic sign was the depressed level of conscience, and the NIHSS ranged from 4 to 12 points.

Despite a wide variety of radiological findings in hospitalized cases (19), it is uncommon for patients to be diagnosed with COVID-19 using brain magnetic resonance imaging (MRI). Microhemorrhages, IS and ICHs are the most prevalent presentations. Other less common findings include hypoxic anoxic brain injury, encephalitis, acute disseminated encephalomyelitis (ADEM), leukoencephalopathy, transient perivascular inflammation of the carotid artery syndrome (TIPIC), and posterior reversible encephalopathy syndrome (PRES). 6 7 8 44 53 57 58

The ICH group had the highest mortality rate, 7 44 followed by patients with leukoencephalopathy and IS, whereas patients with microhemorrhages or encephalitis as sole neuroimaging findings had the lowest mortality rates. 7 Patients in intensive care unit (ICU) had significantly higher incidences of cerebral microhemorrhages and encephalitis/encephalopathy. 58

COVID-19 patients are also at a higher risk for hemorrhagic conversion of their stroke, accompanied by an increased mortality rate. 23 33 Nonetheless, multicompartmental hemorrhage is the ICH subtype with the highest mortality rate, followed by MFH presentations, while SDH had the lowest mortality rate. 17

Considering patients without COVID-19, lobar hemorrhages are often associated with structural changes such as cerebral amyloid angiopathy, arteriovenous malformations or brain tumors. 59 Independent associated risk factors were anticoagulation, a prior history of IS and APOE e2 or e4 genotype, which had a specific association with lobar ICH. 60

Hypertension is the leading attributable risk of non-lobar ICH, followed by prior history of IS and anticoagulation. Interestingly, hypercholesterolemia was less frequent in non-lobar ICH cases. 60 The most common locations of hypertensive ICH are the basal ganglia (caudate nucleus and putamen), thalamus, cerebellum, midbrain, and pons. 59 60

The results of the present review revealed a predominance of hypertension over DM2, dyslipidemia, and other comorbidities. Nevertheless, a distinct proportion of IPH was found, whereas patients had predominantly lobar locations (32%) as opposed to non-lobar (24%). In addition to the association with lobar ICH and ApoE e4e4 allele, recent findings suggest an increased risk of severe COVID-19 infection in this population, independent of preexisting dementia, hypertension, and DM2. 61 62 APOE ε4 carriers also present an increased susceptibility to SARS-CoV-2 infection with higher serum indicators of inflammation. 63

Our case series had an expected predominance of hypertension as comorbidity, considering that all the intraparenchymal hemorrhages were in lobar locations and 40% in non-lobar. Therefore, these findings are consistent with the literature evidence found in this review.

Severe acute respiratory syndrome coronavirus 2-infected stroke patients exhibited particular clinical aspects compared with non-infected patients. The infection was associated with a higher prevalence of younger patients, hemorrhagic conversion of IS, 33 severe NIHSS scores, elevated D-dimer levels, 26 33 64 thrombocytopenia, 33 43 64 elevated PTT, 64 elevated INR, and in-hospital stroke. 43

Considering only ICH, COVID-19 patients were younger with higher rates of malignancies, 25 elevated INR, PTT, and fibrinogen levels, yet decreased frequency of hypertension. 27 No significant changes were reported for other risk factors, such as DM2, dyslipidemia, smoking, ischemic heart disease, or atrial fibrillation. 20 25 26 27 These patients also had more severe NIHSS and ICH scores at admission. 26 27

Although these cases present with higher median neutrophil-to-lymphocyte ratios, there was no significant difference when compared with control groups. 27

The comparison between COVID-19 cases with and without ICH yielded more discrepant results. Those with hemorrhagic events were older and had higher rates of prior stroke, hypertension, DM2, dyslipidemia, congestive heart failure, ischemic heart disease, and smoking, 25 54 but a lower rate of atrial fibrillation 28 and thrombocytopenia. 54 When considering microbleeds alone, the only significant increase was in the rate of disturbance of consciousness prior to MRI, severity of lung computed tomography (CT), days of intubation, and duration of hospital or ICU stay. 18

#### Anticoagulant use

Cohort studies have shown conflicting data on the risk of ICH while in use of therapeutic anticoagulation among patients with and without COVID-19. Some studies report no increased risk of bleeding or mortality, 25 26 33 while others showed a 2 to 7-fold increase in risk of hemorrhagic events 27 28 and a 13-fold higher mortality risk. 27

In the current review, the use of anticoagulation was reported in 43.3% of the 385 patients before diagnosis of ICH, of which 161 patients (47.3%) used therapeutic doses. The prevalence of anticoagulation in cohorts was higher in patients with SAH (86%), followed by MFH (82%), and lower in those with SDH (29%). Some cohort studies reported the use of anticoagulants in 16 to 100% of patients. 17 18 19 20 22 24 25 26 27 28 33 The main indication was for the hypercoagulability of patients with COVID-19, expressed by high levels of D-dimer. 17 27 65 Of all the cohort and case series studies reviewed, alterations in D-dimer were observed in 13 studies, with values ranging from 231 ng/ml to 117,608 ng/mL. The mean value of 10 studies was ∼ 2,912 ng/ml.

### Pathophysiology of ICH in COVID-19 infection

The coronavirus, akin to other respiratory viruses, has neurotropism and the ability to invade the CNS in two ways: hematogenous and retrograde neuronal pathways. This ability to infect neurons from the olfactory bulb can also explain complaints of hyposmia and anosmia. The hematogenous route is identified as the main form of CNS infection, since the virus can infect endothelial capillary cells in the brain or infect leukocytes. Additionally, similarly to SARS-CoV, SARS-CoV-2 exploits the ACE2 receptor for cell entry. 14 66

ACE2 (is a critical enzyme in the renin-angiotensin-aldosterone (RAAS) system that regulates blood pressure, fluid and electrolyte balance, and vascular resistance. This enzyme is extensively expressed in alveolar epithelial cells (type 2 pneumocytes), oral and esophageal mucosa, as well as in vascular endothelial cells, smooth muscle, glial cells, and in some neurons, including those in the cardiorespiratory center of the brainstem. 11 14 67

Severe acute respiratory syndrome coronavirus 2 infection in humans is mediated by S (spike) glycoprotein binding, by the receptor-binding domain (RBD) to ACE2 receptors in host cells, which leads to downregulation of ACE2 expression. This negative regulation during SARS-CoV-2 infection can increase serum levels of angiotensin II, causing endothelial function impairment and blood pressure dysregulation. Therefore, blood pressure fluctuations with an increased risk of hemorrhagic cerebrovascular events can occur. 11 14

The affinity of the SARS-CoV-2 spike protein to ACE2 receptors in brain capillary endothelium can also cause direct vascular injury. The explanation for this involves the process of binding of viral particles by the endothelial cells and, subsequently, damage to the endothelial lining that can cause ruptures and bleeding. This same process can occur within neurons from the viral invasion of the CNS. 11 67

There is a release of cytokines and proteases that accompanies the immune response to SARS-CoV-2 infection, involving massively increased levels of interleukin 6 (IL-6), IL-7, IL-10, IL-1β, interferon-gamma (IFN) -γ), and tumor necrosis factor α (TNF-α), while there is a reduction in CD4 + and CD8 + T cells, indicating that the cytokine storm attenuates adaptive immunity against SARS-CoV infection. 13 68 In critically-ill patients with COVID-19, higher serum levels of inflammatory markers (e.g., C-reactive protein and D-dimers) and an increase in neutrophil-lymphocyte ratio can be seen, also present in the inflammatory process of ICH. 27 69 70

The cytokine storm usually starts in the second week of infection, with the activation of macrophages, dendritic cells, other immune cells, and subsequent massive release of proinflammatory cytokines. 68 Consequently, via a mechanism that is still unclear, changes in the permeability of the BBB can be impaired, facilitating the influx of inflammatory molecules to activate C macrophages and microglia. Ultimately, these cells become hyper-activated and start producing their own set of inflammatory molecules, which can lead to cerebral edema and even hemorrhagic events. 56 68

Thus, BBB breakdown is a possible additional mechanism for several cerebrovascular events associated with this infection, such as hemorrhagic transformation of IS, ICHs, and cases of PRES reported in some patients with COVID-19. 71 72 73

The binding of spike protein may also promote a downregulation of ACE 2 expression in the brain, thereby triggering an increase in local angiotensin II levels and reduction in the vasodilator heptapeptide (angiotensin 1–7). Ang 1–7 acts as a neuroprotective factor by stimulating the release of prostaglandin and nitric oxide, as well as inhibiting the growth of smooth muscle cells and action of catecholamines. 67 74

Patients with hypertension normally have low ACE2 expression, which is further reinforced with SARS-CoV-2 infection, increasing the risk of stroke. 75 The intrinsic relationship between systolic BP variability and poor prognosis of cerebral hemorrhage should be pointed out, as a high variation in BP during the first 24 hours of admission was associated with an unfavorable hospital prognosis in patients with ICH. The lack of BP control might be explained by autonomic dysfunction, with sympathetic predominance, associated with the production of proinflammatory cytokines, hyperglycemia, and increased permeability of the BBB, which are present in SARS Cov2 infection. 75 76

Diabetic patients with COVID-19 are at increased risk of serious complications. The possible mechanisms that lead to an increased risk of stroke in these patients include excessive proinflammatory responses and reduced ACE2 expression by advanced glycosylation, leading to increases in angiotensin I and II. 77

Coagulation disorders may be a plausible hypothesis to explain how SARS-CoV-2 infection can induce brain hemorrhage, as patients with COVID-19 may suffer from consumption coagulopathy with prolonged prothrombin time and reduced fibrinogen, both of which also contribute to secondary cerebral hemorrhage. 75

The older population has several aggravating factors for the development of intravascular hemorrhages, such as cerebral microembolism, white matter lesions, vascular basement membrane thickening, and increased BBB permeability, which promote endothelial damage, changes in elasticity, and subsequent fluctuations in blood flow and pressure causing loss of self-regulation and increase in ICH risk. 78

## Study limitations

The main limitation of this review was the lack of complete data from the majority of articles in the literature. Especially in relation to data from laboratory tests, in-hospital outcomes, and rehabilitation. Furthermore, some studies failed to report details of the statistical method, which imposed difficulty to standardize a measure of central tendency. In this way, as the COVID-19 pandemics is a recent object of study, the overall quality and details of the studies could have been compromised by the urge to provide enlightenment about clinical manifestations of COVID-19.

In conclusion, despite the unusual association, the combination of these two diseases is associated with high rates of mortality and morbidity, as well as more severe clinical-radiological presentations. Further studies are needed to provide robust evidence on the exact pathophysiology behind the occurrence of intracranial hemorrhages after COVID-19 infection.

## References

[1] World Health Organization Coronavirus disease (COVID-19) outbreak [Internet] 2021[cited 2021 Mar 24]. Available from:https://www.who.int/emergencies/diseases/novel-coronavirus-2019

[2] Thakur V Ratho R K Kumar P Multi-Organ Involvement in COVID-19: Beyond Pulmonary Manifestations J Clin Med 2021 10 03 446 33498861 10.3390/jcm10030446 PMC7866189

[3] Sun B W Zhang M Wang P C Song J T A Clinical Analysis of Extrapulmonary Complications in Novel Coronavirus Pneumonia Patients Int J Gen Med 2021 14 381 385 33603443 10.2147/IJGM.S293972 PMC7881799

[4] Mao L Jin H Wang M Neurologic Manifestations of Hospitalized Patients With Coronavirus Disease 2019 in Wuhan, China JAMA Neurol 2020 77 06 683 690 32275288 10.1001/jamaneurol.2020.1127 PMC7149362

[5] Tawakul A A Alharbi A H Basahal A M Neurological Symptoms and Complications of COVID-19 Among Patients in a Tertiary Hospital in Saudi Arabia Cureus 2021 13 11 e19200 34873535 10.7759/cureus.19200 PMC8639190

[6] Chowdhary A Subedi R Tandon M Relevance and clinical significance of magnetic resonance imaging of neurological manifestations in covid-19: A systematic review of case reports and case series. Vol. 10, Brain Sciences MDPI AG 2020 1 15 10.3390/brainsci10121017 PMC7766893 33371260

[7] RSNA COVID-19 task force Mogensen M A Wangaryattawanich P Hartman J Filippi C G Hippe D S Cross N M Special report of the RSNA COVID-19 task force: systematic review of outcomes associated with COVID-19 neuroimaging findings in hospitalized patients Br J Radiol 2021 94 1127 2.0210149E7 10.1259/bjr.20210149 PMC8553187 33914618

[8] Mahammedi A Ramos A Bargalló N Brain and lung imaging correlation in patients with COVID-19: Could the severity of lung disease reflect the prevalence of acute abnormalities on neuroimaging? A global multicenter observational study AJNR Am J Neuroradiol 2021 42 06 1008 1016 33707278 10.3174/ajnr.A7072 PMC8191655

[9] Kuo W Hӓne C Mukherjee P Malik J Yuh E L Expert-level detection of acute intracranial hemorrhage on head computed tomography using deep learning Proc Natl Acad Sci U S A 2019 116 45 22737 22745 31636195 10.1073/pnas.1908021116 PMC6842581

[10] Naidech A M Intracranial hemorrhage Am J Respir Crit Care Med 2011 184 09 998 1006 22167847 10.1164/rccm.201103-0475CI PMC3361326

[11] Baig A M Khaleeq A Ali U Syeda H Evidence of the COVID-19 Virus Targeting the CNS: Tissue Distribution, Host-Virus Interaction, and Proposed Neurotropic Mechanisms ACS Chem Neurosci 2020 11 07 995 998 32167747 10.1021/acschemneuro.0c00122

[12] Klingenstein M Klingenstein S Neckel P H Evidence of SARS-CoV2 Entry Protein ACE2 in the Human Nose and Olfactory Bulb Cells Tissues Organs 2020 209(4-6):155 164 33486479 10.1159/000513040 PMC7900466

[13] Pedersen S F Ho Y C SARS-CoV-2: a storm is raging J Clin Invest 2020 130 05 2202 2205 32217834 10.1172/JCI137647 PMC7190904

[14] Wang Z Yang Y Liang X COVID-19 Associated Ischemic Stroke and Hemorrhagic Stroke: Incidence, Potential Pathological Mechanism, and Management Front Neurol 2020 11 571996 33193019 10.3389/fneur.2020.571996 PMC7652923

[15] Tang N Li D Wang X Sun Z Abnormal coagulation parameters are associated with poor prognosis in patients with novel coronavirus pneumonia J Thromb Haemost 2020 18 04 844 847 32073213 10.1111/jth.14768 PMC7166509

[16] Valderrama E V Humbert K Lord A Frontera J Yaghi S Severe Acute Respiratory Syndrome Coronavirus 2 Infection and Ischemic Stroke Stroke 2020 51 07 e124 e127 32396456 10.1161/STROKEAHA.120.030153

[17] Altschul D J Unda S R de La Garza Ramos R Hemorrhagic presentations of COVID-19: Risk factors for mortality Clin Neurol Neurosurg 2020 198 106112 32738585 10.1016/j.clineuro.2020.106112 PMC7382923

[18] Lersy F Willaume T Brisset J C Critical illness-associated cerebral microbleeds for patients with severe COVID-19: etiologic hypotheses J Neurol 2021 268 08 2676 2684 33219827 10.1007/s00415-020-10313-8 PMC7679237

[19] Rothstein A Oldridge O Schwennesen H Do D Cucchiara B L Acute Cerebrovascular Events in Hospitalized COVID-19 Patients Stroke 2020 51 09 e219 e222 32684145 10.1161/STROKEAHA.120.030995 PMC7386677

[20] John S Hussain S I Piechowski-Jozwiak B Clinical characteristics and admission patterns of stroke patients during the COVID 19 pandemic: A single center retrospective, observational study from the Abu Dhabi, United Arab Emirates Clin Neurol Neurosurg 2020 199 106227 33011516 10.1016/j.clineuro.2020.106227 PMC7485577

[21] Al-Mufti F Becker C Kamal H Acute Cerebrovascular Disorders and Vasculopathies Associated with Significant Mortality in SARS-CoV-2 Patients Admitted to The Intensive Care Unit in The New York Epicenter J Stroke Cerebrovasc Dis 2021 30 02 105429 33276301 10.1016/j.jstrokecerebrovasdis.2020.105429 PMC7605750

[22] Migdady I Shoskes A Hasan L Z Timing of Acute Stroke in COVID-19-A Health System Registry Study Neurohospitalist 2021 11 04 285 294 34567388 10.1177/1941874420985983 PMC8442157

[23] Siegler J E Cardona P Arenillas J F Cerebrovascular events and outcomes in hospitalized patients with COVID-19: The SVIN COVID-19 Multinational Registry Int J Stroke 2021 16 04 437 447 32852257 10.1177/1747493020959216 PMC7533468

[24] Pavlov V Beylerli O Gareev I Torres Solis L F Solís Herrera A Aliev G COVID-19-Related Intracerebral Hemorrhage Front Aging Neurosci 2020 12 600172 33192492 10.3389/fnagi.2020.600172 PMC7642875

[25] Qureshi A I Baskett W I Huang W Intracerebral Hemorrhage and Coronavirus Disease 2019 in a Cohort of 282,718 Hospitalized Patients Neurocrit Care 2022 36 01 259 265 34231186 10.1007/s12028-021-01297-y PMC8260011

[26] Hernández-Fernández F Sandoval Valencia H Barbella-Aponte R A Cerebrovascular disease in patients with COVID-19: neuroimaging, histological and clinical description Brain 2020 143 10 3089 3103 32645151 10.1093/brain/awaa239 PMC7454411

[27] Kvernland A Kumar A Yaghi S Anticoagulation use and Hemorrhagic Stroke in SARS-CoV-2 Patients Treated at a New York Healthcare System Neurocrit Care 2021 34 03 748 759 32839867 10.1007/s12028-020-01077-0 PMC7444897

[28] Melmed K R Cao M Dogra S Risk factors for intracerebral hemorrhage in patients with COVID-19 J Thromb Thrombolysis 2021 51 04 953 960 32968850 10.1007/s11239-020-02288-0 PMC7511245

[29] Abbas R El Naamani K Sweid A Intracranial Hemorrhage in Patients with Coronavirus Disease 2019 (COVID-19): A Case Series World Neurosurg 2021 154 e473 e480 34298138 10.1016/j.wneu.2021.07.067 PMC8294594

[30] Fayed I Pivazyan G Conte A G Chang J Mai J C Intracranial hemorrhage in critically ill patients hospitalized for COVID-19 J Clin Neurosci 2020 81 192 195 33222915 10.1016/j.jocn.2020.08.026 PMC7434499

[31] Keller E Brandi G Winklhofer S Large and Small Cerebral Vessel Involvement in Severe COVID-19: Detailed Clinical Workup of a Case Series Stroke 2020 51 12 3719 3722 33054673 10.1161/STROKEAHA.120.031224 PMC7678671

[32] Mousa-Ibrahim F Berg S Od TPDetola O Teitcher M Ruland S Intracranial Hemorrhage in Hospitalized SARS-CoV-2 Patients: A Case Series J Stroke Cerebrovasc Dis 2021 30 01 105428 33161349 10.1016/j.jstrokecerebrovasdis.2020.105428 PMC7605803

[33] Ramos A D Koyfman F Byrns K Characterization of Hemorrhagic and Ischemic Stroke in a Diverse Cohort of COVID-19 Patients Neurohospitalist 2021 11 04 295 302 34567389 10.1177/1941874421990545 PMC8442153

[34] Sedova P Brown R D Jr Bryndziar T Treat covid-19, but not only covid-19: Stroke matters as well Cerebrovasc Dis 2022 51 01 52 59 34515067 10.1159/000517968 PMC8450853

[35] SSNAP Collaboration Douiri A Muruet W Bhalla A Stroke Care in the United Kingdom During the COVID-19 Pandemic Stroke 2021 52 06 2125 2133 33896223 10.1161/STROKEAHA.120.032253 PMC8140645

[36] Wu Y Chen F Wang Z Reductions in Hospital Admissions and Delays in Acute Stroke Care During the Pandemic of COVID-19 Front Neurol 2020 11 584734 33250851 10.3389/fneur.2020.584734 PMC7674953

[37] Libruder C Ram A Hershkovitz Y Reduction in Acute Stroke Admissions during the COVID-19 Pandemic: Data from a National Stroke Registry Neuroepidemiology 2021 55 05 354 360 34237727 10.1159/000516753 PMC8339012

[38] Balestrino M Coccia A Boffa A S Request of hospital care dropped for TIA but remained stable for stroke during COVID-19 pandemic at a large Italian university hospital Intern Emerg Med 2021 16 03 735 739 33063236 10.1007/s11739-020-02522-w PMC7561243

[39] Diegoli H Magalhães P SC Martins S CO Decrease in Hospital Admissions for Transient Ischemic Attack, Mild, and Moderate Stroke During the COVID-19 Era Stroke 2020 51 08 2315 2321 32530738 10.1161/STROKEAHA.120.030481 PMC7302100

[40] Abdulazim A Ebert A Etminan N Szabo K Alonso A Negative Impact of the COVID-19 Pandemic on Admissions for Intracranial Hemorrhage Front Neurol 2020 11 584522 33071955 10.3389/fneur.2020.584522 PMC7530817

[41] Nogueira R G Abdalkader M Qureshi M M Global impact of COVID-19 on stroke care Int J Stroke 2021 16 05 573 584 33459583 10.1177/1747493021991652 PMC8010375

[42] Tong X King S MC Asaithambi G COVID-19 Pandemic and Quality of Care and Outcomes of Acute Stroke Hospitalizations: the Paul Coverdell National Acute Stroke Program Prev Chronic Dis 2021 18 E82 34410906 10.5888/pcd18.210130 PMC8388201

[43] Katz J M Libman R B Wang J J Cerebrovascular Complications of COVID-19 Stroke 2020 51 09 e227 e231 32757751 10.1161/STROKEAHA.120.031265 PMC7467046

[44] Jain R Young M Dogra S COVID-19 related neuroimaging findings: A signal of thromboembolic complications and a strong prognostic marker of poor patient outcome J Neurol Sci 2020 414 116923 32447193 10.1016/j.jns.2020.116923 PMC7236667

[45] Mendes A Herrmann F R Genton L Incidence, characteristics and clinical relevance of acute stroke in old patients hospitalized with COVID-19 BMC Geriatr 2021 21 01 52 33446113 10.1186/s12877-021-02006-2 PMC7807227

[46] Katsanos A H Palaiodimou L Zand R The Impact of SARS-CoV-2 on Stroke Epidemiology and Care: A Meta-Analysis Ann Neurol 2021 89 02 380 388 33219563 10.1002/ana.25967 PMC7753413

[47] Syahrul S Maliga H A Ilmawan M Hemorrhagic and ischemic stroke in patients with coronavirus disease 2019: incidence, risk factors, and pathogenesis - a systematic review and meta-analysis F1000 Res 2021 10 34 10.12688/f1000research.42308.1 PMC7934095 33708378

[48] Nannoni S de Groot R Bell S Markus H S Stroke in COVID-19: A systematic review and meta-analysis Int J Stroke 2021 16 02 137 149 33103610 10.1177/1747493020972922 PMC7859578

[49] Tan Y K Goh C Leow A ST COVID-19 and ischemic stroke: a systematic review and meta-summary of the literature J Thromb Thrombolysis 2020 50 03 587 595 32661757 10.1007/s11239-020-02228-y PMC7358286

[50] Cheruiyot I Sehmi P Ominde B Intracranial hemorrhage in coronavirus disease 2019 (COVID-19) patients Neurol Sci 2021 42 01 25 33 10.1007/s10072-020-04870-z PMC7605899 33140308

[51] Cagnazzo F Arquizan C Derraz I Neurological manifestations of patients infected with the SARS-CoV-2: a systematic review of the literature J Neurol 2021 268 08 2656 2665 33125542 10.1007/s00415-020-10285-9 PMC7597753

[52] Nawabi J Morotti A Wildgruber M Clinical and imaging characteristics in patients with SARS-CoV-2 infection and acute intracranial hemorrhage J Clin Med 2020 9 08 1 15 10.3390/jcm9082543 PMC7464657 32781623

[53] Büttner L Bauknecht H C Fleckenstein F N Neuroimaging Findings in Conjunction with Severe COVID-19 Röfo Fortschr Geb Röntgenstr Neuen Bildgeb Verfahr 2021 193 07 822 829 33535257 10.1055/a-1345-9784

[54] Shahjouei S Naderi S Li J Risk of stroke in hospitalized SARS-CoV-2 infected patients: A multinational study EBioMedicine 2020 59 102939 32818804 10.1016/j.ebiom.2020.102939 PMC7429203

[55] Cezar-Junior A B Faquini I V Silva J LJ Subarachnoid hemorrhage and COVID-19: Association or coincidence? Medicine (Baltimore) 2020 99 51 e23862 33371170 10.1097/MD.0000000000023862 PMC7748374

[56] Mishra S Choueka M Wang Q Intracranial Hemorrhage in COVID-19 Patients J Stroke Cerebrovasc Dis 2021 30 04 105603 33484980 10.1016/j.jstrokecerebrovasdis.2021.105603 PMC7831866

[57] Sawlani V Scotton S Nader K COVID-19-related intracranial imaging findings: a large single-centre experience Clin Radiol 2021 76 02 108 116 33023738 10.1016/j.crad.2020.09.002 PMC7491990

[58] Choi Y Lee M K Neuroimaging findings of brain MRI and CT in patients with COVID-19: A systematic review and meta-analysis Eur J Radiol 2020 133 109393 33161199 10.1016/j.ejrad.2020.109393 PMC7606068

[59] de Oliveira Manoel A L Goffi A Zampieri F G The critical care management of spontaneous intracranial hemorrhage: a contemporary review Crit Care 2016 20 01 272 27640182 10.1186/s13054-016-1432-0 PMC5027096

[60] Martini S R Flaherty M L Brown W M Risk factors for intracerebral hemorrhage differ according to hemorrhage location Neurology 2012 79 23 2275 2282 23175721 10.1212/WNL.0b013e318276896f PMC3542348

[61] Kuo C L Pilling L C Atkins J L APOE e4 genotype predicts severe COVID-19 in the UK biobank community cohort. Vol. 75, Journals of Gerontology - Series A Biological Sciences and Medical Sciences Oxford University Press 2020 2231 2232 10.1093/gerona/glaa131 PMC7314139 32451547

[62] FinnGen Kurki S N Kantonen J Kaivola K APOE ε4 associates with increased risk of severe COVID-19, cerebral microhaemorrhages and post-COVID mental fatigue: a Finnish biobank, autopsy and clinical study Acta Neuropathol Commun 2021 9 01 199 34949230 10.1186/s40478-021-01302-7 PMC8696243

[63] Zhang H Shao L Lin Z APOE interacts with ACE2 inhibiting SARS-CoV-2 cellular entry and inflammation in COVID-19 patients Signal Transduct Target Ther 2022 7 01 261 https://www.nature.com/articles/s41392-022-01118-4[Internet] 35915083 10.1038/s41392-022-01118-4 PMC9340718

[64] Mount Sinai Stroke Investigators* Dhamoon M S Thaler A Gururangan K Acute Cerebrovascular Events With COVID-19 Infection Stroke 2021 52 01 48 56 33280551 10.1161/STROKEAHA.120.031668

[65] Dogra S Jain R Cao M Hemorrhagic stroke and anticoagulation in COVID-19 J Stroke Cerebrovasc Dis 2020 29 08 104984 32689588 10.1016/j.jstrokecerebrovasdis.2020.104984 PMC7245254

[66] Paniz-Mondolfi A Bryce C Grimes Z Central nervous system involvement by severe acute respiratory syndrome coronavirus-2 (SARS-CoV-2) J Med Virol 2020 92 07 699 702 32314810 10.1002/jmv.25915 PMC7264598

[67] Wang H Tang X Fan H Potential mechanisms of hemorrhagic stroke in elderly COVID-19 patients Aging (Albany NY) 2020 12 11 10022 10034 32527987 10.18632/aging.103335 PMC7346040

[68] Perrin P Collongues N Baloglu S Cytokine release syndrome-associated encephalopathy in patients with COVID-19 Eur J Neurol 2021 28 01 248 258 32853434 10.1111/ene.14491 PMC7461405

[69] Zhang F Ren Y Shi Y Predictive ability of admission neutrophil to lymphocyte ratio on short-term outcome in patients with spontaneous cerebellar hemorrhage Medicine (Baltimore) 2019 98 25 e16120 31232961 10.1097/MD.0000000000016120 PMC6636913

[70] Lattanzi S di Napoli M Ricci S Divani A A Matrix Metalloproteinases in Acute Intracerebral Hemorrhage. Vol. 17, Neurotherapeutics Springer 2020 484 496 10.1007/s13311-020-00839-0 PMC7283398 31975152

[71] Dakay K Kaur G Mayer S A Santarelli J Gandhi C Al-Mufti F Cerebral Herniation Secondary to Stroke-Associated Hemorrhagic Transformation, Fulminant Cerebral Edema in Setting of COVID-19 Associated ARDS and Active Malignancy J Stroke Cerebrovasc Dis 2020 29 12 105397 33096499 10.1016/j.jstrokecerebrovasdis.2020.105397 PMC7547646

[72] Dias D A de Brito L A Neves L O Paiva R GS Barbosa Júnior O A Tavares-Júnior J WL Hemorrhagic PRES: an unusual neurologic manifestation in two COVID-19 patients Arq Neuropsiquiatr 2020 78 11 739 740 33331467 10.1590/0004-282X20200184

[73] Princiotta Cariddi L Tabaee Damavandi P Carimati F Reversible Encephalopathy Syndrome (PRES) in a COVID-19 patient J Neurol 2020 267 11 3157 3160 32583053 10.1007/s00415-020-10001-7 PMC7312113

[74] Sweid A Hammoud B Bekelis K Cerebral ischemic and hemorrhagic complications of coronavirus disease 2019 Int J Stroke 2020 15 07 733 742 32501751 10.1177/1747493020937189 PMC7534206

[75] Wang H Y Li X L Yan Z R Sun X P Han J Zhang B W Potential neurological symptoms of COVID-19 Ther Adv Neurol Disord 2020 13 1.75628642091783E15 10.1177/1756286420917830 PMC7119227 32284735

[76] Divani A A Liu X Di Napoli M Blood Pressure Variability Predicts Poor In-Hospital Outcome in Spontaneous Intracerebral Hemorrhage Stroke 2019 50 08 2023 2029 31216966 10.1161/STROKEAHA.119.025514

[77] Pal R Bhansali A COVID-19, diabetes mellitus and ACE2: The conundrum Diabetes Res Clin Pract 2020 162 108132 32234504 10.1016/j.diabres.2020.108132 PMC7118535

[78] Camacho E LoPresti M A Bruce S The role of age in intracerebral hemorrhages J Clin Neurosci 2015 22 12 1867 1870 26375325 10.1016/j.jocn.2015.04.020

