# Supplementary material for: Intracranial hemorrhages in patients with COVID-19: a systematic review of the literature, regarding six cases in an Amazonian population
Source: Arq Neuropsiquiatr. 2023 Nov 30;81(11):989–99. doi: 10.1055/s-0043-1772834 (PMC10689113; doi:10.1055/s-0043-1772834)
Supplement: Supplementary file 1 — Supplementary Material [file 10-1055-s-0043-1772834-s220230.pdf]

Supplementary Table 1 Characteristics of the included cohorts and case series studies

Author	Study design	No. of events	Sex n (%)	Median (IQR), or mean ± SD	Comorbidity	Antithrombotic use	Type of ICH	Location of hemorrhage	Intervention	Outcome	mRS % (n)
Siegler 2021	Cohort	28	Male 18 (65%)	NA	NA	NA	IPH 71.4% ($n=20$)<br>HC 50% ($n=14$)<br>SAH 25% ($n=7$)<br>IVH 28.6% ($n=8$)<br>SDH 7.1% ($n=2$)	Supratentorial ($n=13$)<br>Infratentorial ($n=7$)	NA	Mortality 57.1% ($n=16$)	NA
Shahjouei 2020	Cohort	27	Male 21 (77%)	62 (52.5–71.5)	NA	NA	IPH 92% ($n=25$)<br>SAH 7.4% ($n=2$)	NA	NA	NA	NA
Rothstein 2020	Cohort	8	Male 4 (50%)	57/7	Hypertension 75% ($n=6$)<br>Dyslipidemia 63% ($n=5$)<br>DM2 38% ($n=3$)<br>CAD 38% ($n=3$)<br>Obesity 38% ($n=3$)<br>Prior stroke 13% ($n=1$)	Anticoagulation 88% ($n=7$)<br>Anticoagulation + antiplatelet 50% ($n=4$)	IPH 63% ($n=5$)<br>SAH 38% ($n=3$)<br>SDH 12.5% ($n=1$)<br>MFH 12.5% ($n=1$)<br>MCH 12.5% ($n=1$)	IPH: (Supratentorial ($n=5$):<br>Lobar $n=5$))<br>SAH: Cortical ($n=3$)<br>MCH: IPH/SAH/SDH	Intensive care with mechanical ventilation 75% ($n=6$)	Mortality 75% ($n=6$)<br>VTE (38%) ($n=3$)<br>MI (12.5%) ($n=1$)	NA
Migdady 2021	Cohort	3	Male 2 (67%)	56.6 (38–70)	Hypertension 100% ($n=3$) Dyslipidemia 67% ($n=2$) DM2 67% ($n=2$) Cancer 33% ($n=1$)	Antiplatelets 100% ($n=3$)	IPH 100% ($n=3$)<br>IVH 33% ($n=2$)	IPH: Supratentorial ($n=3$): Subinsular ($n=1$) Frontal ($n=1$) Parietal ($n=1$)	Intensive care with mechanical ventilation 100% ($n=3$)	Mortality 67% ($n=2$)<br>Discharge to rehabilitation 33% ($n=1$)	3–5 33% ($n=1$)<br>6 66% ($n=2$)
Pavlov 2020	Cohort	3	Male 3 100%	60 (56–64)	Hypertension 100% ($n=3$)<br>Smoking 66.6% ($n=2$) DM2 33.3% ($n=1$)<br>Dyslipidemia 33.3% ($n=1$)	Prophylactic anticoagulation 33% ($n=1$)	IPH 100% ($n=3$)<br>IVH 66% ($n=2$)	IPH: Supratentorial ($n=3$): Lobar ($n=1$)<br>Basal ganglia ($n=2$)	Intensive care with mechanical ventilation 33% ($n=1$)<br>Decompressive craniectomy 66% ($n=2$)	Intensive care with multiple organ failure 33% ($n=1$)<br>Discharge to rehabilitation 66% ($n=2$)	0–3 33% ($n=1$)<br>4–6 66% ($n=2$)
Qureshi 2022	Cohort	154	Male 103 (66.9%)	62.1/17.2	Hypertension 81.8% ($n=126$)<br>Dyslipidemia 50.6% ($n=78$)<br>DM2 49.4% ($n=76$) AF 31.8% ($n=49$) CHF 24.7% ($n=38$)<br>Smoking 20.8% ($n=32$)<br>Prior ICH 18.2% ($n=28$) Alcoholism 8.4% ($n=13$)	NA	SAH 11.7% ($n=18$)	NA	EVD 7,1% ($n=11$)<br>Invasive ICP monitoring 0.6% ($n=1$)	Mortality 40.3% ($n=62$)<br>Non-routine discharge* 43.5% ($n=67$)<br>Discharged to home 16.2% ($n=25$)	NA
John 2020	Cohort	12	Male 10 (83.3%)	48.1/13.3	Hypertension 58.3% ($n=7$) DM2 25% ($n=3$) Smoking 8.3% ($n=1$) Dyslipidemia 8.3% ($n=1$)	Anticoagulation (16.7%) ($n=2$)	SAH 33% ($n=4$)	NA	EVD 33.3% ($n=4$)<br>Endovascular treatment (-coiling/embolization) 16.7% ($n=2$)	Mortality 16.7% ($n=2$)<br>Discharge to Home 41.6% ($n=5$).<br>Hospital transference 16.7% ($n=2$).	0–2 50% ($n=6$)<br>3–5 33.3% ($n=4$)<br>6 16.7% ($n=2$)
Hernández-Fernández 2020	Cohort	5	Male 4 (80%)	62.6/7.2	Hypertension 80% ($n=4$) Dyslipidemia 60% ($n=3$) DM2 40% ($n=2$) Smoking 20% ($n=1$)	Therapeutical anticoagulation 60% ($n=3$)	IPH 100% ($n=5$)<br>FICH 80% ($n=4$)<br>MCH 80% ($n=4$)<br>SAH 80% ($n=4$)<br>MFH 20% ($n=1$)<br>Microbleeds 20% ($n=1$)<br>IVH 20% ($n=1$)	IPH: Supratentorial ($n=5$): Lobar ($n=4$)<br>Basal Ganglia ($n=1$)<br>Infratentorial ($n=1$)<br>Superficial ($n=3$) Deep ($n=1$)<br>MCH: IPH/SAH ($n=3$)<br>IPH/IVH/SAH ($n=1$)	Decompressive craniectomy 60% ($n=3$)<br>ICP monitoring 20% ($n=1$)	Mortality 40% ($n=2$)	0–3 0 (0)<br>4–6 100% ($n=5$)
Lersy 2021	Cohort	19	Male 16 (84%)	66 (44–79)	Hypertension 37% ($n=7$) Dyslipidemia 37% ($n=7$) Smoking 21% ($n=4$) Prior stroke 16% ($n=3$) DM2 11% ($n=2$)	Therapeutical anticoagulation 100% ($n=19$)	Microbleeds 100% ($n=19$) MFH 100% ($n=19$) SAH 26%	IPH: Isolated ($n=3$)<br>Multiple ($n=1$)	Intensive care with mechanical ventilation 100% ($n=19$)	Mortality (21%) ($n=4$)<br>Hospital discharge (37%) ($n=7$)	NA

 © 2023. The Author(s).

Supplementary Table 1 (*Continued*)

Author	Study design	No. of events	Sex n (%)	Median (IQR), or mean ± SD	Comorbidity	Antithrombotic use	Type of ICH	Location of hemorrhage	Intervention	Outcome	mRS % (n)
							(*n* = 5) IPH 21% (*n* = 4)				
Kvernland 2021	Cohort	19	Male 15 (79%)	60 (51–63)	Hypertension 42.1% (*n* = 8) Dyslipidemia 36.8% (*n* = 7) DM2 31.6% (*n* = 6)<br>AF 5.2% (*n* = 1)	Therapeutical anticoagulation 89.5% (*n* = 17) Supratherapeutic 58.8% (10/17). In the therapeutic range 29.4% (5/17)	IPH 52.6% (*n* = 13)<br>FICH 63% (*n* = 12)<br>SAH 42% (*n* = 8)<br>MFH = 37% (*n* = 7).<br>IVH 37% (*n* = 7)<br>MCH 47.3% (*n* = 5)<br>Microbleed 15.7% (*n* = 3)	IPH: Supratentorial 77% (*n* = 8): Lobar (*n* = 6) Infratentorial 22% (*n* = 2)<br>Superficial 31.5% (*n* = 5) Deep 10% (*n* = 2)<br>MCH: IPH/IVH/SAH (*n* = 5)	Intensive care with mechanical ventilation 84.2% (*n* = 16)	Mortality In-hospital 84.6% (11/13)<br>Still hospitalized 31% (6/19)<br>Discharge to home 7% (1/13)<br>Discharge to rehabilitation 7% (1/13)	NA
Altschul 2020	Cohort	35	Male 21 (60%)	67.03/15.5	Hypertension 71% (*n* = 25) DM2 28.6% (*n* = 10) CHF 17.1% (*n* = 6) Cancer 14.3% (*n* = 2) Previous MI 5.7% (*n* = 2)	Anticoagulation 20% (*n* = 7)<br>Antiplatelets 14.3% (*n* = 5)	SDH 48.5% (*n* = 17) MCH =20% (*n* = 7) FICH = 14.2% (*n* = 5)<br>MFH = 11.4% (*n* = 4) SAH 5.7% (*n* = 2)	NA	Surgical management 11.4% (*n* = 4)	General mortality 45.7% (*n* = 16):<br>SDH 35.3% (*n* = 6)<br>SAH 50% (*n* = 1)<br>MCH 71.4% (*n* = 5)<br>MFH 50% (*n* = 2)<br>FICH 40%(*n* = 2).<br>Non-routine discharge* 91.4% (*n* = 32).	NA
Katz 2020	Cohort	29	NA	NA	NA	NA	HC 53.3% (*n* = 6) (100% Ischemic stroke)	NA	NA	NA	NA
Melmed 2021	Cohort	33	Male 26 (78%)	61.6	Hypertension 66.6% (*n* = 22)<br>DM2 36.4% (*n* = 12)<br>AF 27.3% (*n* = 9) CAD 12.1% (*n* = 4)	Therapeutical anticoagulation 72.7% (*n* = 24)	NA	NA	NA	Mortality 51.5% (*n* = 17)	NA
Ramos 2021	Cohort	11	NA	NA	NA	Therapeutical anticoagulation 9% (*n* = 1)	IPH 72% (*n* = 8) HC 36.3% (*n* = 4) (100% Ischemic stroke)<br>MCH 18% (*n* = 2)	IPH: Superficial 36.3% (*n* = 4) Deep 18.1% (*n* = 2)	EVD 9% (*N* = 1)	NA	NA
Sawlani 2021	Cohort	17	NA	NA	NA	NA	Microbleeds70% (*n* = 12)<br>AHNE 11% (*n* = 2)<br>IPH 11% (*n* = 2)<br>SAH 5% (*n* = 1)	IPH: Supratentorial: Lobar (*n* = 2).<br>Microbleeds: Parenquimal (*n* = 12) Callosal (*n* = 4)	NA	NA	NA
Salahuddin 2020	Cohort	1	NA	NA	NA	NA	SAH 100% (*N* = 1)	SAH: Pseudoaneurysm (*n* = 1): PICA	NA	NA	NA
Dhamoon 2021	Cohort	20	NA	NA	NA	NA	IPH 80% (*n* = 16)<br>SAH 20% (*n* = 4)	IPH: Supratentorial:<br>Frontal 56.3% (*n* = 9)<br>Temporal 25% (*n* = 4)<br>Parietal 31% (*n* = 5)<br>Occipital 25% (*n* = 4)<br>Basal ganglia 31%	NA	NA	NA

(*Continued*)

Supplementary Table 1 (*Continued*)

Author	Study design	No. of events	Sex n (%)	Median (IQR), or mean ± SD	Comorbidity	Antithrombotic use	Type of ICH	Location of hemorrhage	Intervention	Outcome	mRS % (n)
								($n=5$) Infratentorial 12.5% ($n=2$)			
Al-Mufti 2021	Cohort	2	Male 1 50%	56 (31–81)	NA	NA	SAH 100% ($N=2$)	SAH: Arterial dissection of ICA ($n=1$) MCA and vertebral artery ($n=1$)	NA	NA	NA
Abbas 2021	Case Series	19	Male 11 (57.9%)	52.6/15.8	Hypertension 52.6% ($n=10$) CHF 31.6% ($n=6$) Dyslipidemia 21.1% ($n=4$) DM2 21.1% ($n=4$) CKD 15.8% ($n=3$) CHD 15.8% ($n=3$) CLD 5.3% ($n=1$) AF15.8% ($n=3$) Smoking 5.3% ($n=1$)	Therapeutical anticoagulation 21% ($n=4$)	IPH 63.1% ($n=12$) SAH 31.6% ($n=6$) IVH 15.7% ($n=3$) SDH 5.3% ($n=1$)	IPH: Supratentorial: Lobar ($n=4$) Thalamic ($n=2$) Infratentorial: cerebellar ($n=1$) IVH ($n=3$) SAH: Spontaneous non-aneurysmal ($n=2$) Aneurysm ($n=4$): PICA ($n=1$) AcA ($n=1$)	Decompressive craniectomy (13.3%) ($n=2$) Aneurysm embolization (20%) ($n=3$) Supportive (66.7%) ($n=10$)	Mortality 52.6% ($n=10$) Discharge to Home 66.7% ($n=4$) Discharge to rehabilitation 33.3% ($n=2$)	($n=17$) 0 11.8% ($n=2$) 1 5.9% ($n=1$) 2 5.9% ($n=1$) 3 11.8%($n=2$) 4 0 (0) 5 5.9%($n=5$)
Nawabi 2020	Case Series	18	Male 9 (50%)	49.5	Hypertension 55.6% ($n=10$) DM2 22.2% ($n=4$)	Anticoagulation 44% ($n=8$) Antiplatelets 5.4% ($n=1$)	SAH (61%) ($n=11$) IPH 33.3% ($n=6$) IVH (16.7% ($n=3$) MCH 11% ($n=2$) SDH 5.6% ($n=1$)	IPH: Supratentorial: ($n=1$) Lobar ($n=4$) Deep ($n=1$)/ Infratentorial ($n=1$) SAH: Cortical $n=9$ MCH: SDH/SAH ($n=1$) IPH/SAH ($n=1$)	Decompressive craniectomy (5.6%) ($n=1$) EVD (5.6%) ($n=1$) Intensive care with mechanical ventilation (72%) ($n=13$)	Mortality 44.4% ($n=8$) Multiple organ Failure 44.4% ($n=8$)	0–3 0 (0) 4–6 (100%) ($n=18$)
Keller 2020	Case Series	6	NA	NA	Hypertension 100% ($n=6$) DM2 66.6% ($n=4$) Obesity 50% ($n=3$) Dyslipidemia 16.6% ($n=1$) AF 16.6% ($n=1$)	Therapeutical anticoagulation 62% ($n=4$) Prophylactic anticoagulation 37% ($n=2$)	MFH 83% ($n=5$) Microbleed 66% ($n=4$) SAH 50% ($n=3$) FICH 16% ($n=1$) IVH 16% ($n=1$)	SAH: Lobar ($n=2$) Cerebellar ($n=1$)	Intensive care with mechanical ventilation 100% ($n=6$)	Mortality 16% ($n=1$) Still hospitalized 83% ($n=5$)	NA
Parra-Romero 2021	Case Series	4	Male 2 (50%)	71.5 (56–84)	Hypertension 50% ($n=2$) DM2 50% ($n=2$)	Prophylactic anticoagulation 50% ($n=2$)	IPH 100% ($n=4$) IVH 75% ($n=3$) FICH 75% ($n=3$) MFH 25% ($n=1$)	IPH: Infratentorial: Cerebellar ($n=2$) Supratentorial: Thalamic ($n=2$) Deep ($n=4$) Superficial ($n=1$)	NA	Mortality75% ($n=3$) Discharge to home 25% ($n=1$)	NA
Trifan 2020	Case series	19	Male 9 (56%)	64	NA	Anticoagulation 44% ($n=7$)	IPH 84% ($n=16$) FICH 80% ($n=15$) SAH 16% ($n=3$) MFH 5% ($n=1$)	IPH: Supratentorial ($n=12$) Infratentorial 18% ($n=3$)	NA	Mortality 52% ($n=10$) Discharge to Home 10% ($n=2$) Discharge to rehabilitation 10% ($n=2$). Nonroutine discharge* 16% ($n=3$)	mRS 6 (median) 4 (IQR) mRS survivors 2 (median) 1 (IQR)
Cezar-Junior 2020	Case series	4	Female 3 (75%)	55.25 (36–71)	Hypertension 50% ($n=2$) Obesity 25% ($n=1$)	NA	SAH 100% ($n=4$) MCH 25% ($n=1$)	SAH: Saccular aneurysm of PcA ($n=1$) Interhemispheric SDH ($n=1$) MCH: SDH/SAH ($n=1$)	Nimodipine. anti-vasospasm therapy (100%) ($n=4$). EVD (50%) ($n=2$). Balloon-assisted embolization (25%) ($n=1$)	Mortality 25% ($n=1$) Discharged to rehabilitation 50% ($n=2$) Discharge to home 25% ($n=1$)	0–2 25% ($n=1$) 3–5 50% ($n=2$) 6 50% ($n=1$)

 © 2023. The Author(s).

Supplementary Table 1 (*Continued*)

Author	Study design	No. of events	Sex n (%)	Median (IQR), or mean ± SD	Comorbidity	Antithrombotic use	Type of ICH	Location of hemorrhage	Intervention	Outcome	mRS % (n)
Dodd 2021	Case series	10	Female 5 (50%)	38.5 (29–57)	Hypertension 10% (*n* = 1)	NA	SAH 100% (*n* = 10)	SAH: Saccular aneurysm (*n* = 5): AcA (*n* = 2) MCA (*n* = 1) PcA (*n* = 1) OA (*n* = 1) Blister aneurysm (*n* = 1): AcoA pseudoaneurysm (*n* = 4): VA (*n* = 1) AcA (*n* = 1) PICA (*n* = 1) PCA (*n* = 1)	Coiling 40% Flow diversion 30% Clipping 20% Expiration prior to treatment 10%	Mortality 20% (*n* = 2)	0–2 70% (*n* = 7) 3–4 10% (*n* = 1) 5–6 20% (*n* = 2)
Mishra 2021	Case series	8	Male 5 (62.5%)	71 (30–89)	Hypertension 75% (*n* = 6) Obesity 62.5% (*n* = 5) Dyslipidemia 37.5% (*n* = 3) DM2 37.5% (*n* = 3) CKD 12.5% (*n* = 1) Cancer 12.5% (*n* = 1) Prior stroke 12.5% (*n* = 1) Smoking 12.5% (*n* = 1)	Therapeutical anticoagulation 50% (*n* = 4) Antiplaquets 25% (*n* = 2)	SAH 62%% (*n* = 5) IVH 50% (*n* = 4) MCH 50% (*n* = 4) MFH 37.5% (*n* = 3) FICH 62%% (*n* = 5) IPH 87.5% (*n* = 7)	IPH:(Supratentorial (*n* = 5): Lobar (*n* = 2) Thalamic (*n* = 1) Basal ganglia (*n* = 2) Infratentorial (*n* = 2): Cerebellar (*n* = 1) Pontine (*n* = 1)) Deep (*n* = 4) Superficial (*n* = 3) MCH: IPH/ SAH (*n* = 2) IPH/ IVH/SAH (*n* = 2)	NA	Mortality 37.5% (*n* = 3) Discharge to home 12.5% (*n* = 1) Discharge to rehabilitation (50%) (*n* = 4) NIHSS in Discharge 13.2 (3–24) (*n* = 5)	NA
Mousa-Ibrahim 2021	Case Series	5	Female 3 (60%)	68.5 (54 - 79)	DM2 80% (*n* = 4) Hypertension 80% (*n* = 4) Obesity 60% (*n* = 3) Dyslipidemia 40% (*n* = 2) AF 20% (*n* = 1) GERD 20% (n =) CAD 20% (*n* = 1)	Prophylactic anticoagulation 100% (*n* = 5)	MCH 40% (*n* = 2) FICH 40% (*n* = 2) MFH 60% (*n* = 3) SDH 20% (*n* = 1) SAH 40% (*n* = 2) IVH 20% (*n* = 1) IPH 80% (*n* = 4)	IPH: Infratentorial (*n* = 2): Cerebellar (*n* = 2) Supratentorial (*n* = 3): Frontal (*n* = 3) MCH: IPH/IVH/SAH (*n* = 1) IPH/SDH (*n* = 1)	Decompressive craniectomy 20% (*n* = 1) Protamine 20% (*n* = 1)	Mortality 80% (*n* = 4) Discharge to rehabilitation 20% (*n* = 1)	NA

Abbreviations: AcA, anterior communicating artery; AcoA, anterior choroidal artery; AF, atrial fibrillation; AHNE, Acute hemorrhagic necrotizing encephalitis; CAD, coronary artery disease; CHD, chronic hepatic disease; CHF, congestive heart failure; CKD, chronic kidney disease; CLD, chronic lung disease; DM2, diabetes mellitus; EVD, external ventricular drain; FICH, focal intracranial hemorrhage; GERD, gastroesophageal reflux disease; HC, hemorrhagic conversion; ICA, internal carotid artery; ICH, intracranial hemorrhage; ICP, intracranial pressure; IPH, intraparenchymal hemorrhage; IQR, interquartile range; IVH, intraventricular hemorrhage; MCA, middle cerebral artery; MCH, multicompartmental hemorrhage; MFH, multifocal hemorrhage; MI, myocardial infarction; mRS, modified rankin score; NA, not available; No, number; OA, ophthalmic artery; PCA, posterior cerebral artery; PcA, posterior communicating artery; PICA, posterior inferior cerebellar artery; SAH, subarachnoid hemorrhage; SD, standard deviation; SDH, subdural hematoma; VA, vertebral artery; VTE, venous thromboembolism.
Note: Non routine discharge*: discharge to short-term hospital or other facility, including intermediate care and skilled nursing home.

Supplementary Table 2 Characteristics of the individuals presenting Intraparenchymal hemorrhages

Patient (Ref.)	Gender	Age	Initial symptoms onset (days)	Neurologic symptoms	Hemorrhagic presentation	Comorbidity	Antithrombotic prior ICH	Neuroimaging method	Radiologic findings	Intervention	Outcomes
1*	M	66	Respiratory, 15 days	Depressed level of consciousness GCS 05	IPH/IVH	Hypertension	None	CT	Intraventricular findings of hemorrhage with ventricular dilatation and signs of herniation	Measures to control intracranial pressure<br>EVD	Death
2*	F	59	Respiratory, 7 days	Depressed level of consciousness<br>seizures	IPH/IVH	Hypertension	None	CT	Left Lentiform nucleus hemorrhage with signs of ventricular extension.<br>Midline shift with uncal herniation	Measures to control intracranial pressure	Death
43 Urciuoli 2021	M	67	Respiratory, 7 days	Dysarthria and left hemiparesis	IPH	Hypertension	None	CT	HIP in the region of the right basal ganglia with a midline deviation of 3mm	Conservative Treatment	Discharged to acute rehabilitation<br>Incomplete functional improvement
45 Charra 2021	M	61	Respiratory	Persistent depressed level of consciousness after two weeks of decreased sedation	IPH	Recurrent thrombophlebitis	Therapeutic (UFH)	CT	IPH on the rostrum of the corpus callosum	NA	NA
47 Flores 2020	M	40	Respiratory	Depressed level of consciousness (GCS 05) pinpoint pupils complete left hemiparesis intact left corneal, cough, and gag reflexes	IPH/IVH	Hypertension<br>Obesity<br>DM2	None	CT	Extensive pontine and midbrain hemorrhage with intraventricular extension involving the third and fourth ventricles and early hydrocephalus.	EVD with ICP of 10mmHG<br>Palliative care	Multi-organ failure
48 Fatehi 2020	F	50	Respiratory<br>Neurologic	Severe headache with frequent vomiting and depressed level of consciousness	IPH	None	None	CT	IPH in left parietal lobe	Intensive care support	Death
34 Li 2020	M	68	Respiratory, 7 days	On the 19th day of admission for DM2, a diminished state of consciousness was observed subsequent to a reduction in sedation	MCH/MFH/IPH/IVH/SAH	Atrial fibrillation	Therapeutic (LMWH)	CT	ICH in right temporal occipital lobe and left frontal occipital parietal lobe hemorrhage with extension to bilateral lateral ventricles (especially on the right),SAH and brain herniation	Intensive Care support<br>Measures to control intracranial pressure<br>Mannitol	Multi-organ failure<br>Coma<br>Death
36 Srinivasan 2021	M	18	Neurologic, 2 days	Thunderclap headache with nausea and vomiting	MCH/IPH/SAH/RCVS	Asthma	None	CT/ CTA	CTA:Multifocal areas of non-occlusive and bilateral stenosis in M2/M3 middle cerebral artery and P2/P3 posterior cerebral artery segments, as well as right anterior cerebral segment A2<br>CT: Right frontal lobe hemorrhage with	Descompressive Craniectomy<br>Verapamil 80mg IV 8/8h	Discharged to rehabilitation<br>Complete functional improvement

 © 2023. The Author(s).

Supplementary Table 2 (*Continued*)

Patient (Ref.)	Gender	Age	Initial symptoms onset (days)	Neurologic symptoms	Hemorrhagic presentation	Comorbidity	Antithrombotic prior ICH	Neuroimaging method	Radiologic findings	Intervention	Outcomes
									midline shift associated with SAH in frontal and parietal lobes (ICH score = 3)		
38 Krett 2020	M	69	Respiratory, 4 days	Acute confusional state at D13 with depressed level of consciousness and diffuse flaccid paresis with areflexia after decrease of sedation	MCH/MFH/IPH/ SAH<br>Hemorrhagic encephalopathy	Hypertension<br>DM2<br>CAD	None	MRI	MRI: multicompartmental and multifocal hemorrhages with perilesional vasogenic edema	Intensive care support	Discharged to rehabilitation<br>Incomplete functional improvement
39 Soldatelli 2020	M	67	Respiratory, 6 days	At D16 with depressed level of consciousness after decrease of sedation tonic downward gaze deviation	MFH/MCH/IPH/IVH/ SAH/Microbleeds	Hypertension<br>DM2<br>Dyslipidemia	Prophylactic (LMWH)	MRI	SWI: Punctual hypointense lesions in the cortico-cortical junction and corpus callosum, as well as HIP in the left thalamus and posterior internal capsule with drainage to lateral ventricles<br>FLAIR: SAH in the right frontoparietal region	Non-invasive ICP monitoring	Discharged to rehabilitation
40 Fayed 2020	1 F	57	Respiratory	At D20 with persistent encephalopathy after two weeks of intubation	MFH/IPH	Hypertension<br>OSA<br>Obesity<br>Asthma	Heparin drip (hemodialysis	CT/MRI	CT: subacute IPH in the right frontal lobe, measuring 3.5 × 3.4cm with frontal midline deviation<br>MRI: IPH in the left frontal lobe, measuring 0.6 cm (not using heparin) with significant inflammation in the sphenoid sinus	Intensive care support	Discharged to rehabilitation
44 Bao 2020	M	38	Neurologic, 0 days	Sudden loss of consciousness right side miosis with weakened light reflex diparesis with paresthesia right spastic paresis with Babinski sign present	MFH/IPH/IVH	None	None	CT	CT: Extensive IPH in the left temporal lobe, basal ganglia and corona radiata, with maximum size of 8.5 × 4.2 cm. Presence of perilesional edema, compression and extension to the left ventricle Midline shift to the right.	Decompressive craniectomy<br>Intensive care support<br>Measures to control intracranial pressure<br>Mannitol	Incomplete functional improvement

(*Continued*)

 © 2023. The Author(s).

Supplementary Table 2 (*Continued*)

Patient (Ref.)	Gender	Age	Initial symptoms onset (days)	Neurologic symptoms	Hemorrhagic presentation	Comorbidity	Antithrombotic prior ICH	Neuroimaging method	Radiologic findings	Intervention	Outcomes
53 Princiotta Cariddi 2020	F	64	Respiratory, 10 days	At D25 with altered level of consciousness, blurred vision, complete left side hemiparesis and symmetric hyporeflexia after decrease of sedation	MFH/IPH/PRES	Hypertension<br>Atrial fibrillation<br>Dyslipidemia<br>GERD<br>OSA	Aspirin	CT/MRI	CT:Symmetrical bilateral frontal and posterior temporo-parieto-occipital hypodensity of the subcortical white matter and a small left occipital IPH. MRI: vasogenic edema and right temporal hypodensity, correlated to hemorrhagic process.	Intensive care support	Incomplete functional improvement

Abbreviations: CAD, coronary artery disease; CT, computerized tomography; CTA, computed tomography angiography; D, day from admission; DM2, diabetes mellitus; EVD, external ventricular drain; F, female; FLAIR, fluid attenuated inversion recovery; GCS, Glasgow coma scale; GERD, gastroesophageal reflux disease; ICP, intracranial pressure; IPH, intraparenchymal hemorrhage; IVH, intraventricular hemorrhage; LMWH, low molecular weight heparin; M, male; MCH, multicompartmental hemorrhage; MFH, multifocal hemorrhage; MRI, magnetic resonance imaging; NA, not available; OSA, obstructive sleep apnea; PRES, posterior reversible encephalopathy syndrome; RCV, reversible cerebral vasoconstriction syndrome; Ref., reference; SAH, subarachnoid hemorrhage; SWI, susceptibility weighted imaging; UFH, unfractionated heparin.
Note: *Current case.

 © 2023. The Author(s).

Supplementary Table 3 Characteristics of the individuals presenting subdural and subarachnoid hemorrhages

Patient (Ref.)	Gender	Age	Initial symptoms onset (days)	Neurologic symptoms	Hemorrhagic presentation	Comorbity	Antithrombotic prior onset	Neuroimaging method	Radiologic findings	Intervention	Outcomes
3*	M	77	Neurologic, 2 days	Acute confusional state axial and appendicular ataxia NIHSS 4	SDH	Hypertension CKD	None	CT	Subacute SDH with mass effect in right frontal, temporal and parietal lobes Compression of right ventricles and midline deviation	Measures to control ICP Subdural hematoma drainage	Discharged for reahabilitation
52 Al-Olama 2020	M	36	Respiratory, 6 days	Acute confusional state GCS 13	MCH/ SDH/IPH/ SAH	None	None	CT	Right frontal lobe IPH associated with SAH in the ipsilateral sylvian fissure and frontal and temporal lobes Acute right SDH with surrounding edema and causing midline shift. In the post-contrast phase, there was leptomeningeal enhancement on the right, suggesting viral meningoencephalitis	Intensive care support Subdural hematoma drainage	NA
32 Avci 2020	M	50	Respiratory, 7 days	Sudden depressed level of consciousness GCS 04	SAH/IVH	None	None	CT	Bilateral parasagittal SAH in the circle of Willis Extension to the lateral ventricles and the 3rd ventricle	Intensive care support	Death at D83
33 Saiegh 2020	1 M	31	Respiratory, 7 days	Sudden headache with depressed level of consciousness	SAH/IVH	None	None	CT/ CTA	CT: Posterior fossa SAH, including 4th ventricle and with signs of hydrocephalus CTA: ruptured and dissecting aneurysm of PICA artery	Intensive care support EVD+ Flow deviation stent	Discharged to reahabilitation
35 Scheer 2022	M	56	Respiratory, 30 days	Progressive headache with episodes of thunderclap headache Coma at admission GCS 03	SAH/RCVS	None	None	CT/ CTA	CT: Massive SAH in the left frontal lobe with midline shift CTA: No vascular malformation or perfusion alterations. Narrowing of the middle cerebral artery with narrowing and dilatation of the anterior cerebral artery in "beads"	Decompressive Craniectomy	Death

(*Continued*)

Supplementary Table 3 (*Continued*)

Patient (Ref.)	Gender	Age	Initial symptoms onset (days)	Neurologic symptoms	Hemorrhagic presentation	Comorbity	Antithrombotic prior onset	Neuroimaging method	Radiologic findings	Intervention	Outcomes
37 Dakay 2020	F	30	Respiratory, 7 days	Thunderclap headache	SAH/RCVS	Migraine	None	CT/ DSA	CT: bilateral frontal convex SAH<br>DSA: vertebral dissection (left V2 segment) and 7 days later with bilateral anterior circulation vasospasm, suggesting RCVS (RCVS score= 10)	Intensive care support	Discharged to home
40 Fayed 2020	F	54	Respiratory, 6 days	At D8 (postintubation) presented dilated and non-reactive pupils	SAH/IVH	Hypertension<br>Breast cancer	Heparin drip (30 hour)<br>Prophylactic aspirin	CT	Diffuse SAH distributed throughout the basal cisterns with intraventricular extension and sulcal effacement	Intensive care support	Death at D12
46 Ghosh 2022	F	35	Respiratory, 5 days	Thunderclap headache/ Nuchal rigidity<br>GCS 12<br>Minimal bilateral lateral rectus restriction<br>Grade-1 papilledema	SAH	None	None	CT/CTA/DSA	SAH involving basal cisterns, interhemispheric fissure (feathery outlines) and both sylvian fissures<br>CTA/DSA: No evidence of cerebral aneurysms or arteriovenous malformations	Intensive care support<br>Nimodipine	Discharged to home

Abbreviations: CKD, chronic kidney disease; CT, computerized tomography; CTA, computed tomography angiography; D, day from admission; DSA, digital subtraction angiography; EVD, external ventricular drain; F, female; GCS, Glasgow coma scale; ICP, intracranial pressure; IPH, intraparenchymal hemorrhage; IVH, intraventricular hemorrhage; M, male; MCH, multicompartmental hemorrhage; MRI, magnetic resonance imaging; NA, not available; NIHSS, NIH stroke scale; PICA, posterior inferior cerebellar artery; RCVS, reversible cerebral vasoconstriction syndrome; Ref., reference; SAH, subarachnoid hemorrhage; SDH, subdural hemorrhage.
Note: *Current case.

 © 2023. The Author(s).

Supplementary Table 4 Characteristics of the individuals presenting hemorrhagic conversion

Patient (Ref.)	Gender	Age	Initial symptoms onset (days)	Neurologic symptoms	Hemorrhagic presentation	Comorbidity	Antithrombotic prior onset	Neuroimaging method	Radiologic findings	Intervention	Outcomes
4*	F	39	Respiratory, 16 days	Headache	Hemorrhagic conversion (CVT)/ IPH	Breast cancer	None	CT	Superior sagittal sinus and left transverse sinus thrombosis<br>Rope sign indicating cortical vein thrombosis	Anticoagulation	Death
5*	F	42	Neurologic, 0 days	Complete right hemiparesis<br>Headache<br>NIHSS 4	Hemorrhagic conversion (CVT)/ IPH	None	None	CT/MRI	Ischemic areas in right frontal and parietal lobes signs of hemorrhage in MRI gradient echo sequence Thrombosis of superior sagittal sinus and right transverse sinus, as well as in cortical veins	Anticoagulation	Discharged to home<br>mRS 0 NIHSS 1
6*	M	71	Respiratory, 23 days	Complete left hemiparesis<br>NIHSS 12	Hemorrhagic conversion (IS)/ IPH	Hypertension<br>DM2	Thrombolytic Therapy	CT	Ischemic area in the right temporoparietal region with grade 1 hemorrhagic transformation after thrombolytic therapy	Thrombolytic Therapy	Discharged to home<br>mRS 1 NIHSS 0
33 AlSaiegh 2020	2 F	62	Neurologic, 0 days	Sudden onset of right hemiparesis and aphasia	Hemorrhagic conversion (IS)/ IPH	None	Thrombolytic therapy	CT/CTA/MRI	CTA: Left middle cerebral artery occlusion at PO10 showed hemorrhagic conversion with midline shift and obstructive hydrocephalus	Mechanical thrombectomy decompressive hemicraniectomy + EVD	NA
40 Fayed 2020	3 M	71	Respiratory, 0 days	At D20 with Persistent encephalopathy after two weeks of intubation	Hemorrhagic conversion (IS)/IPH	Hypertension<br>DM2<br>CKD<br>Cel B lymphoma	Thrombolytic therapy	CT	Right occipital ICH with surrounding edema suggestive of hemorrhagic conversion of na infarct	Palliative care with extubation	Multi-organ failure

(*Continued*)

 © 2023. The Author(s).

Supplementary Table 4 (*Continued*)

Patient (Ref.)	Gender	Age	Initial symptoms onset (days)	Neurologic symptoms	Hemorrhagic presentation	Comorbidity	Antithrombotic prior onset	Neuroimaging method	Radiologic findings	Intervention	Outcomes
41 Rajdev 2020	M	62	Respiratory, 12 days	At D10 with paraplegia on minimal sedation	Hemorrhagic conversion (IS)/ MCH/ IPH/ SAH	Hypertension Smoking	Thrombolytic therapy	CT	Subacute ischemic stroke in right occipital lobe, associated with right IPH midline shift with compression of the lateral ventricle and scattered SAH	Palliative Care	Death
42 Owolabi 2021	1 M	59	Respiratory, 5 days	4-hour history of sudden right-side incoordination, altered level of consciousness (GCS 13), Complete right hemiparesis with abducent palsy and subtle neck stiffness	Hemorrhagic conversion (IS)/MFH/ IPH/ IVH	None	Thrombolytic Therapy	CT	Extensive left acute fronto-parietal subcortical infarct with hemorrhagic transformation and bilateral ventricular extension	Intensive care support	Death
	2 M	51	Neurologic, 2 days	Sudden complete left hemiparesis GCS 15	Hemorrhagic Conversion (IS)/ IPH/ Microbleed	None	Thrombolytic Therapy	CT	Small-sized right hemispheric infarct in basal ganglia with a pin-point hemorrhagic transformation.	Intensive care support	Functional improvement
49 Beretta 2021	F	62	Respiratory, 21 days	Moderate agitation with global aphasia<br>Right-sided neglect<br>Severe right hemiparesis	Hemorrhagic conversion (CVT)/ SAH	Hypertension	None	CT/ CT Venography	CT: a left parietal hypodense lesion and a sulcal SAH over the left temporal lobe.<br>CT venography: massive CVT, involving the right transverse sinus, the right jugular bulb, the superior sagittal sinus, the straight sinus, the vein of Galen, and both internal cerebral veins.	Intensive care support<br>Anticoagulation (UFH and LMWH)	Discharged to Rehabilitation

 © 2023. The Author(s).

Supplementary Table 4 (*Continued*)

Patient (Ref.)	Gender	Age	Initial symptoms onset (days)	Neurologic symptoms	Hemorrhagic presentation	Comorbidity	Antithrombotic prior onset	Neuroimaging method	Radiologic findings	Intervention	Outcomes
50 Dakay 2021	1 M	26	Respiratory, 14 days	Complete left hemiparesis with mild sensory loss NIHSS 6	Hemorrhagic conversion (CVT)/ IPH	None	None	CT/MRV/ Angiography	CT: Right parasagittal IPH<br>MRV: did not show any dural venous thrombosis<br>Angiography: Extensive dural venous sinus thrombosis involving the straight sinus, torcula, left transverse and sigmoid sinus, extending into the jugular vein, as well as right transverse sinus, superior sagittal sinus and left vein of Labbe.	Intensive care support	Discharged to acute rehabilitation<br>Incomplete functional improvement
51 Khazaei 2021	M	57	Respiratory, 21 days	Headache followed by left hemiplegia<br>Two episodes of generalized tonic clonic seizures<br>Depressed level of consciousness GCS 02 (unresponsive and mid-dilated pupils)	Hemorrhagic conversion (CVT)/ MCH/IPH/SAH	None	None	CT/MRI/MRV	CT: Right transverse sinus and cortical veins thrombosis with SAH due to hemorrhagic transformation<br>IPH in the middle and posterior right temporal lobe inconsistent with the previous finding<br>MRI/MRV: Confirmed findings of CT	Anticoagulation (UFH)	Discharged to home<br>Complete functional improvement

Abbreviations: CKD, chronic kidney disease; CT, computerized tomography; CTA, computed tomography angiography; CVT, cerebral vein thrombosis; D, day from admission; DM2, diabetes mellitus; EVD, external ventricular drain; F, female; GCS, Glasgow coma scale; ICH, intracranial hemorrhage; ICP, intracranial pressure; IPH, intraparenchymal hemorrhage; IS, ischemic stroke; IVH, intraventricular hemorrhage; LMWH, low molecular weight heparin; M, male; MCH, multicompartmental hemorrhage; MFH, multifocal hemorrhage; MRI, magnetic resonance imaging; mRS, modified Rankin score; MRV, magnetic resonance venography; NA, not available; NIHSS, NIH stroke scale; PO, post-operation day; Ref., reference; SAH, subarachnoid hemorrhage; UFH, unfractionated heparin.
Note: *Current case.

 © 2023. The Author(s).
